# XIAP promotes metastasis of bladder cancer cells by ubiquitylating YTHDC1

**DOI:** 10.1038/s41419-025-07545-9

**Published:** 2025-03-25

**Authors:** Ning Sun, Sijia Wang, Jianting Liu, Peipei Zhang, Yixin Chang, Hongyan Li, Kun Zhao, Yijie Liu, Mingzhi Huang, Yan Hu, Zhenni Lin, Yongyong Lu, Guosong Jiang, Wei Chen, Chuanshu Huang, Honglei Jin

**Affiliations:** 1 Zhejiang Provincial Key Laboratory of Medical Genetics, Key Laboratory of Laboratory Medicine, Ministry of Education, School of Laboratory Medicine and Life Sciences, Wenzhou Medical University, Wenzhou, Zhejiang China; 2 Department of Urology, The First Affiliated Hospital of Wenzhou Medical University, Wenzhou, Zhejiang China; 3 Department of Urology, Union Hospital, Tongji Medical College, Huazhong University of Science and Technology, Wuhan, China

**Keywords:** Cell invasion, Ubiquitin ligases

## Abstract

X-linked inhibitor of apoptosis protein (XIAP), a member of the IAP family, is overexpressed in a variety of tumors and plays an important role in tumor progression. Increasing evidence suggests that XIAP promotes metastasis of bladder cancer but the underlying mechanism is not very clear. The RNA N6-methyladenosine (m^6^A) reader YTHDC1 regulates RNA splicing, nuclear transport, and mRNA stability and is a potential tumor target; however, its ubiquitin E3 ligase has not been described. In this study, screening of proteins that specifically interact with XIAP identified YTHDC1 as its degradation substrate. Ectopic overexpression of XIAP promoted degradation of YTHDC1, and knockout of XIAP upregulated YTHDC1, which inhibited metastasis of bladder cancer. Furthermore, YTHDC1 reduced the expression of matrix metalloproteinase-2 (MMP-2) by destabilizing its mRNA. These experiments revealed that XIAP promotes ubiquitination of YTHDC1, positively regulating expression of the MMP-2 and promoting metastasis of bladder cancer. Collectively, these findings demonstrate that XIAP is a critical regulator of YTHDC1 and pinpoint the XIAP/YTHDC1/MMP-2 axis as a promising target for the treatment of bladder cancer.

## Introduction

Bladder cancer is one of the most common malignant tumors in the urinary system. These tumors are classified as non-muscle-invasive bladder cancer (NMIBC) or muscle-invasive bladder cancer (MIBC) according to whether or not the tumor cells have invaded the muscle layer of the bladder [1]. When the tumor enters the muscle, treatment becomes difficult and the 5-year survival rate is significantly reduced [2]. At present, MIBC is treated by surgery combined with radiotherapy and chemotherapy. However, the prognosis continues to be poor, so there is an urgent need for further treatment modalities. With the emergence of tumor-targeted therapy, the exploration of novel tumor markers and molecular mechanisms may be essential for the treatment of patients with bladder cancer.

X-linked inhibitor of apoptosis protein (XIAP) is the strongest inhibitor of apoptosis in the IAP family and exerts its inhibitory effect by directly binding and inhibiting caspases [3–5]. XIAP consists of four major domains: three N-terminal BIR domains (BIR1-3) and the C-terminal RING zinc finger domain, which is the key structure of XIAP involved in protein modification [6, 7]. For example, XIAP ubiquitinates MDM2 to regulate p53 and inhibit autophagy [8]. XIAP also promotes the growth of breast and colon cancer by promoting p62 depletion through ubiquitination-dependent proteasome degradation and binds directly to Cdc42 for proteasomal degradation [9, 10]. As a ubiquitin E3 ligase, XIAP is an important protein in tumors. Our previous studies have shown that XIAP can promote bladder metastasis by regulating MMP-2, MMP-9, or Rho-GDIβ [11–13], but the direct binding targets are not clear. Identification of substrates for XIAP could help us to understand how XIAP is involved in the development of bladder cancer and help to develop targeted therapies. Therefore, we have a research interest in the substrate of XIAP and its mechanism.

N6-methyladenosine (m^6^A) is a type of epigenetics that has received extensive attention in recent years. The whole dynamic process requires methyltransferase (writer), demethyltransferase (eraser), and reading (reader) proteins for completion [14]. Several studies have demonstrated that m^6^A-related enzyme disorders lead to abnormal modification of m^6^A and aberrant gene expression. FBW7 mediates the proteolytic degradation of YTHDF2 in ovarian cancer [15], and UBC13 promotes ubiquitination and nuclear translocation of FTO [16]. Although XIAP is known to promote substrate ubiquitination, it is unclear whether it regulates m^6^A-related enzymes.

YTHDC1, a member of the YTH family of proteins, can specifically recognize and bind m^6^A-containing RNAs [17–19]. YTHDC1 play an important role in decisions regarding cell fate, including mRNA splicing, export and stability [20–22]. YTHDC1 abnormalities have recently been noted in not only germline development but also human malignancies, including bladder cancer, lung cancer, and breast cancer [23–25]. YTHDC1 inhibits progression of bladder cancer by reducing glycolysis. Moreover, overexpression of YTHDC1 promotes sensitivity to cisplatin by stabilizing PTEN mRNA [26]. YTHDC2 is an RNA helicase and a newly discovered member of the YTH family of proteins, but many of its functions remain unclear [27, 28]. Like other YTH proteins, YTHDC2 is involved in development of lung cancer, gastric cancer, and colorectal cancer [29–31]. However, the current research is focused mainly on the function of m^6^A, and little is known about how expression of m^6^A-related enzymes is regulated. Clarification of the underlying molecular mechanism that regulates modification of m^6^A would provide a novel perspective with regard to the treatment of bladder cancer.

This study aimed to identify the direct downstream targets of XIAP regulation and the upstream regulation mechanism of YTHDC1. We identified an XIAP/YTHDC1/MMP-2 pathway that promotes metastasis of bladder cancer. Our findings indicate that the ubiquitin E3 ligase XIAP promotes metastasis of bladder cancer cells by degrading YTHDC1.

## Results

### XIAP promoted metastasis of bladder cancer

**Fig. 1:**
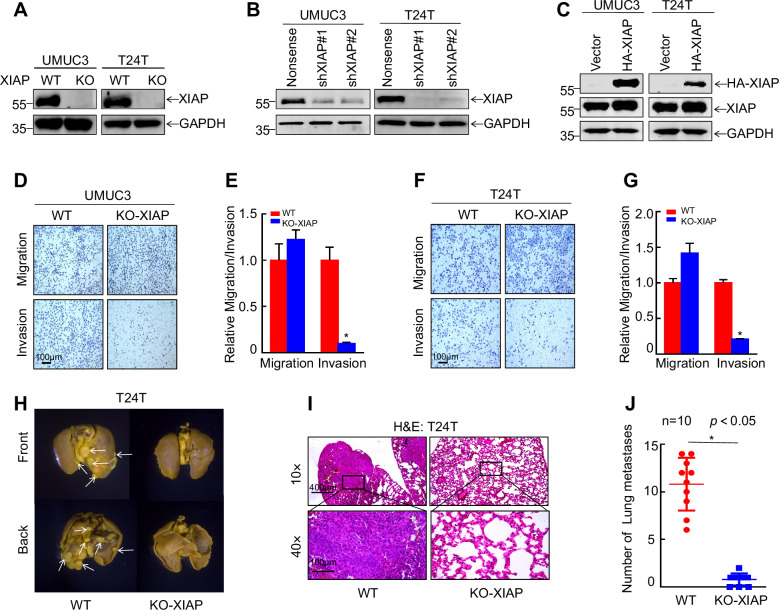
XIAP promotes bladder cancer metastasis. UMUC3/T24T with XIAP knockout (**A**), XIAP knockdown (**B**) and XIAP overexpression (**C**). Transwell assay was used to detect the effect of XIAP knockout on the migration and invasion ability of UMUC3 (**D**) and T24T (**F**) in vitro. The statistical results of migration and invasion ability of UMUC3 (**E**) and T24T (**G**). **H** T24T (WT-XIAP) and T24T (KO-XIAP) cells was injected into nude mice through the tail vein. The formation of lung metastases was observed 12 weeks later. **I** Paraffin embedding of lung tissue for HE staining analysis. **J** The number of lung metastases were counted. Data are represented as mean ± SD and *p < 0.05 between groups.

XIAP is upregulated in patients with bladder cancer and in mouse models of BBN-induced muscle-invasive bladder cancer and can promote metastasis of bladder cancer cells, as found in our previous research [12, 13]. Knockdown of XIAP can inhibit invasion of bladder cancer cells and formation of lung metastasis in vivo. However, the molecular mechanism by which XIAP promotes metastasis of bladder cancer remains unclear. We constructed a model of XIAP knockout and knockdown and XIAP overexpression in UMUC3 and T24T cell lines to investigate the direct downstream target of XIAP in promotion of metastasis of bladder cancer cells (Fig. 1A–C). Transwell assay was then used to confirm the ability of the cells to metastasize in vitro. This assay consistently showed that knockout of XIAP markedly inhibited invasion of bladder cancer cells in vitro (Fig. 1D–G). In order to further confirmed that XIAP knockout influence on lung metastases, we injected wild-type T24T cells and T24T (KO-XIAP) cells into BALB/c-nude mice. XIAP knockout dramatically reduced the number of lung metastases (Fig. 1H–J). These findings confirmed that XIAP promotes invasion of bladder cancer in vitro and in vivo and were consistent with our previous findings.

### XIAP downregulated expression of the tumor suppressor YTHDC1

**Fig. 2:**
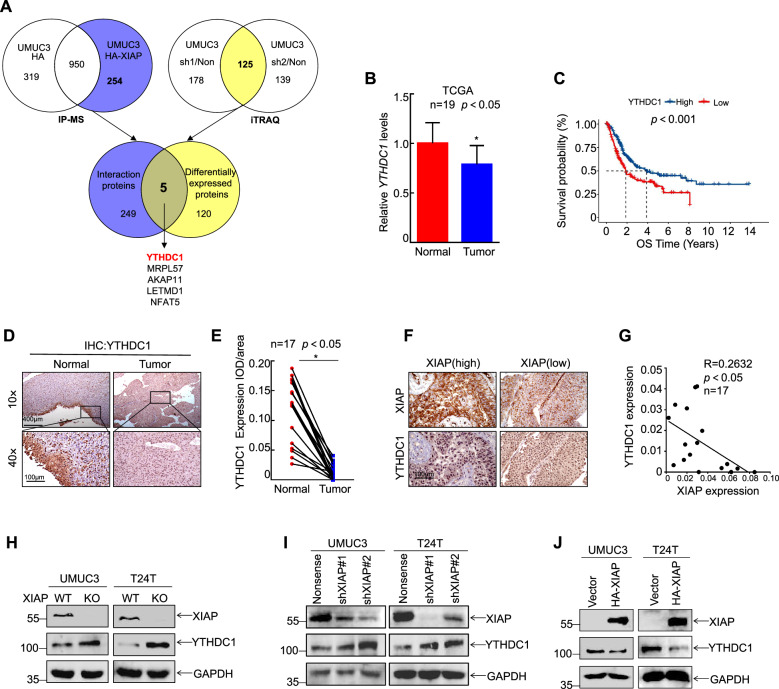
XIAP downregulated the expression of tumor suppressor YTHDC1. **A** The downstream targets of XIAP were analyzed by IP mass spectrometry and iTRAQ proteomics. **B** The TCGA database was used to analyze the differential expression of YTHDC1 in clinical bladder cancer samples. **C** Overall survival of YTHDC1 high expression group and low expression group. **D**, **E** IHC was used to analyze the differential expression of YTHDC1 protein in clinical bladder cancer samples. **F**, **G** The correlation of XIAP with YTHDC1 protein levels were analyzed. Data were analyzed using Pearson correlation. Western Blot was used to detect the protein expression levels of YTHDC1 after XIAP knockout (**H**), knockdown (**I**) or overexpression (**J**) in UMUC3/T24T cells.

Immunoprecipitation-mass spectrometry (IP-MS) and iTRAQ were performed to identify the direct targets of XIAP in bladder cancer and the mechanisms involved. After ectopic overexpression of XIAP in UMUC3 cells and pull-down with anti-HA beads, IP-MS was used to detect proteins that interact with XIAP. Next, we identified proteins that were differentially expressed after knockdown of XIAP by iTRAQ. We intersected the two sets of data and identified YTHDC1, MRPL57, AKAP11, LETMD1, and NFAT5 as potential downstream targets that both interact with and are regulated by XIAP (Fig. 2A). We then investigated data for 19 pairs of patients with bladder cancer in The Cancer Genome Atlas database (TCGA) and found that expression levels of YTHDC1 and AKAP11 in bladder cancer tissue were downregulated in comparison with those in adjacent normal tissues (Figs. 2B and S1A-D). However, iTRAQ identified that the increase in YTHDC1 was more prominent than that of AKAP11 after knockout of XIAP. Moreover, overall survival in patients with bladder cancer was significantly longer in those with higher YTHDC1 expression than in those with low YTHDC1 expression (Fig. 2C). Immunohistochemical analysis of specimens from the 17 pairs of patients with bladder cancer showed that YTHDC1 expression was markedly lower in bladder cancer tissue than in adjacent normal tissue (Fig. 2D, E). Furthermore, the data indicated a negative correlation between XIAP and YTHDC1 in tumor samples (Fig. 2F, G). We also examined the expression of YTHDC1 in bladder cancer cells with knockout, knockdown, and overexpression of XIAP by western blotting. We found that knockout and knockdown of XIAP resulted in elevated expression of YTHDC1 (Fig. 2H, I). In contrast, overexpression of XIAP decreased YTHDC1 protein levels in these cells (Fig. 2J). Overall, these findings demonstrated that YTHDC1 acts as tumor suppressor in bladder cancer. We then hypothesized that YTHDC1 may have an important role in regulation of XIAP during metastasis of bladder cancer.

### XIAP promoted invasion of bladder cancer cells by inhibition of expression of YTHDC1

**Fig. 3:**
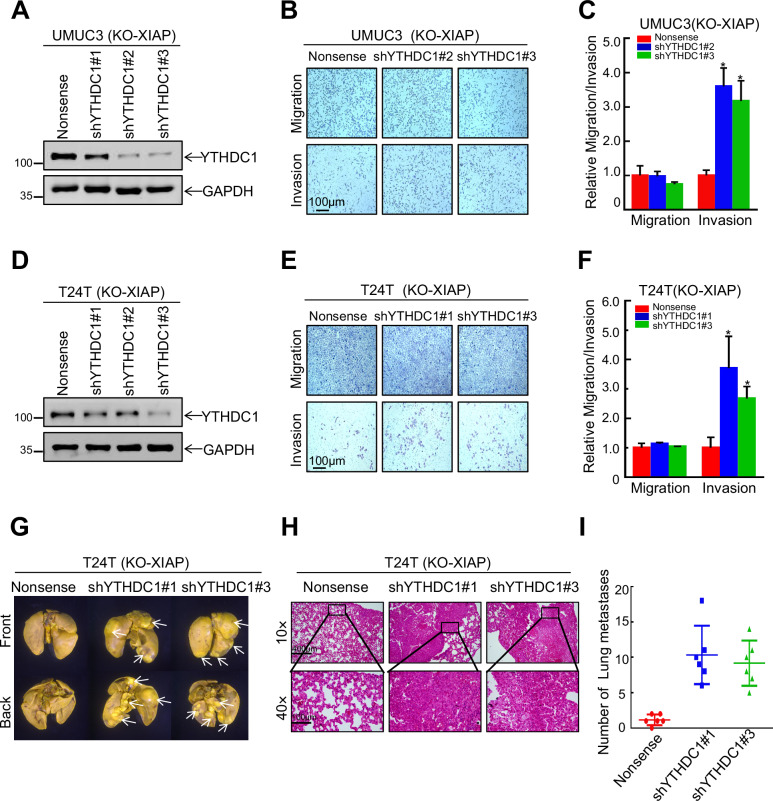
XIAP promotes the invasion of bladder cancer cells by inhibiting the expression of YTHDC1. **A**, **D** Plasmids nonsense, shYTHDC1#1, shYTHDC1#2 and shYTHDC1#3 were packed with lentivirus to infect UMUC3 (KO-XIAP) (**A**) and T24T (KO-XIAP) (**D**), and the knockdown efficiency was detected by western blotting. Transwell assay was used to detect the effects of YTHDC1 knockdown on migration and invasion ability of UMUC3 (KO-XIAP) (**B**) and T24T (KO-XIAP) (**E**). The statistical results of migration and invasion ability of YTHDC1 knockdown in UMUC3 (KO-XIAP) (**C**) and T24T (KO-XIAP) (**F**). **G** T24T (KO-XIAP Nonsense), T24T (KO-XIAP shYTHDC1#1) and T24T (KO-XIAP shYTHDC1#3) cells were injected into nude mice through the tail vein. The formation of lung metastases was observed 12 weeks later. **H** Paraffin embedding of lung tissue for HE staining analysis. **I** The number of lung metastases was counted.

YTHDC1 is known to act as a tumor suppressor in bladder cancer and to affect expression of downstream genes by modification of m^6^A. However, there are no reports on the involvement of XIAP in modification of m^6^A. To confirm whether XIAP promotes metastasis of bladder cancer cells via an effect on YTHDC1, we transfected YTHDC1 knockdown and control plasmids into UMUC3 (KO-XIAP), T24T (KO-XIAP), and wild-type cells to construct stable cells (Figs. 3A, D and S2A, B). Transwell assays confirmed that knockdown of YTHDC1 in UMUC3 (KO-XIAP), T24T (KO-XIAP) and wild-type cells markedly reversed invasion of bladder cancer cells in comparison with control cells in vitro (Figs. 3B, C, E, F and S2C–F). We also injected T24T (KO-XIAP/nonsense), T24T (KO-XIAP/shYTHDC1#1), and T24T (KO-XIAP/shYTHDC1#3) cells into BALB/c-nude mice and observed formation of lung metastases. Counting the number of lung metastases and using hematoxylin-eosin staining, we found that lung metastasis was strongly promoted by knockdown of YTHDC1 (Fig. 3G–I). These findings suggested that XIAP promotes invasion of bladder cancer cells by downregulating expression of YTHDC1.

### XIAP regulated ubiquitination and stability of YTHDC1

**Fig. 4:**
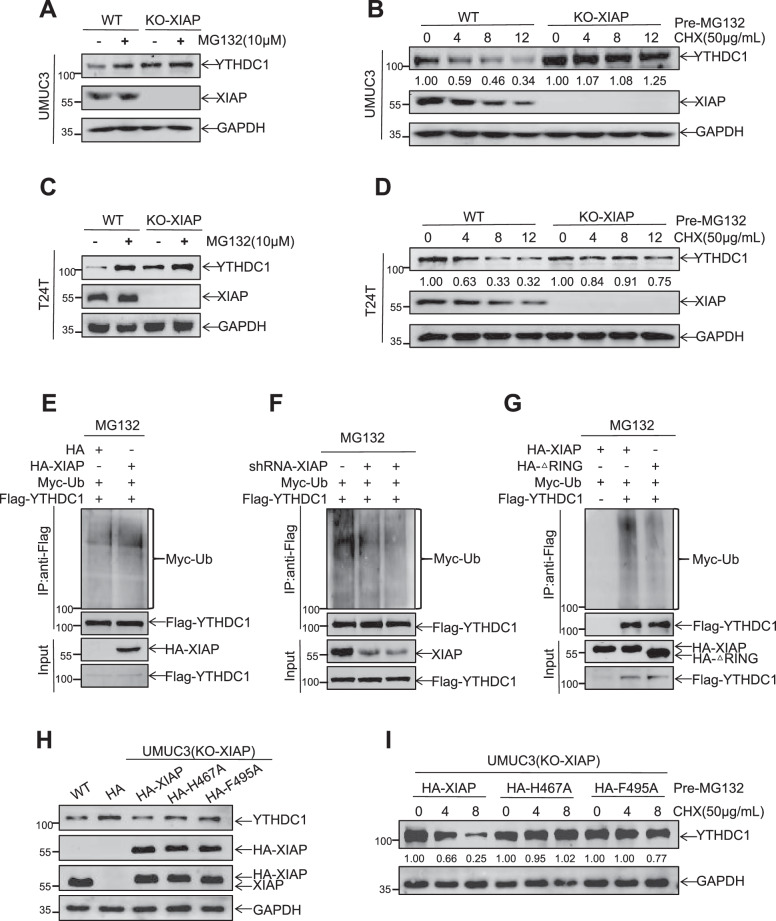
XIAP regulates YTHDC1 ubiquitination and stability. UMUC3 (KO-XIAP), T24T (KO-XIAP) and control cells were pretreated with MG132 (10 μM) for 8 h (**A**, **C**), CHX (50 μg/mL) was then added at 4, 8, and 12 h and the degradation rate of YTHDC1 was detected by western blotting (**B**, **D**). **E** Ubiquitination assays to detect the effects of XIAP overexpression on YTHDC1 ubiquitination levels in UMUC3 cells. **F** Ubiquitination assays to detect the effects of XIAP knockdown on YTHDC1 ubiquitination levels in UMUC3 cells. **G** Ubiquitination assays to detect the effects of RING domain deletion of XIAP on YTHDC1 ubiquitination levels in UMUC3 cells. **H** YTHDC1 expression was detected after overexpression of XIAP-WT, 467 and 495 mutant plasmids by western blotting in UMUC3 (KO-XIAP) cells. **I** Detection of YTHDC1 degradation rate in HA-XIAP, HA-XIAP_H467A_ and HA-XIAP_F495A_ recovered UMUC3 (KO-XIAP) cells.

Next, we investigated the molecular mechanism underlying regulation of YTHDC1 by XIAP. We tested the mRNA levels of YTHDC1 and found that knockdown of XIAP had no effect on YTHDC1 mRNA in UMUC3 cells or T24T cells (Fig. S3A, B). To confirm whether or not the effect of XIAP on YTHDC1 expression is mediated by protein modification, UMUC3/T24T (KO-XIAP) and wild-type cells were treated with the proteasome inhibitor MG132 (10 μM) to impede degradation of protein (Fig. 4A, C). The cells were then treated with the protein synthesis inhibitor cycloheximide (50 μg/mL) for 4, 8, and 12 h, after which expression of YTHDC1 was detected using western blotting. Notably, degradation of YTHDC1 was significantly slower in cells with knockout of XIAP than in wild-type cells (Fig. 4B, D). XIAP is an E3 ligase with a RING domain that increases protein ubiquitination, including MDM2, p62, and Cdc42 [8–10]. Therefore, we performed an in vitro ubiquitination assay to confirm whether or not YTHDC1 ubiquitination levels are affected by XIAP. As expected, the ubiquitination level of YTHDC1 increased in response to ectopic overexpression of XIAP and decreased when XIAP was knocked down (Fig. 4E, F). Furthermore, deletion of the RING domain in XIAP reduced the level of ubiquitination of YTHDC1 (Fig. 4G). These findings suggested that XIAP promotes degradation of YTHDC1 protein by increasing ubiquitination levels.

Previous studies have found that XIAP with mutations at amino acids 467 or 495 does not have intrinsic ubiquitin E3 ligase activity [32, 33]. Therefore, to determine whether XIAP affects the ubiquitination of YTHDC1, we ectopically expressed HA-XIAP, HA-XIAP_H467A_, and HA-XIAP_F495A_ plasmids in UMUC3 (KO-XIAP) cells. We found that mutants XIAP_H467A_ or XIAP_F495A_ failed to suppress YTHDC1 levels while restoration of HA-XIAP still inhibited expression of YTHDC1 in UMUC3 (KO-XIAP) cells (Fig. 4H). We also treated UMUC3 cells with MG132 and cycloheximide for 4 h and 8 h, then measured degradation of YTHDC1. Compared with HA-XIAP, mutant XIAP_H467A_ or XIAP_F495A_ inhibited the rate of degradation of YTHDC1 (Fig. 4I). These data indicated that XIAP promotes ubiquitination and degradation of YTHDC1.

### XIAP is a novel ubiquitin E3 ligase for YTHDC1

**Fig. 5:**
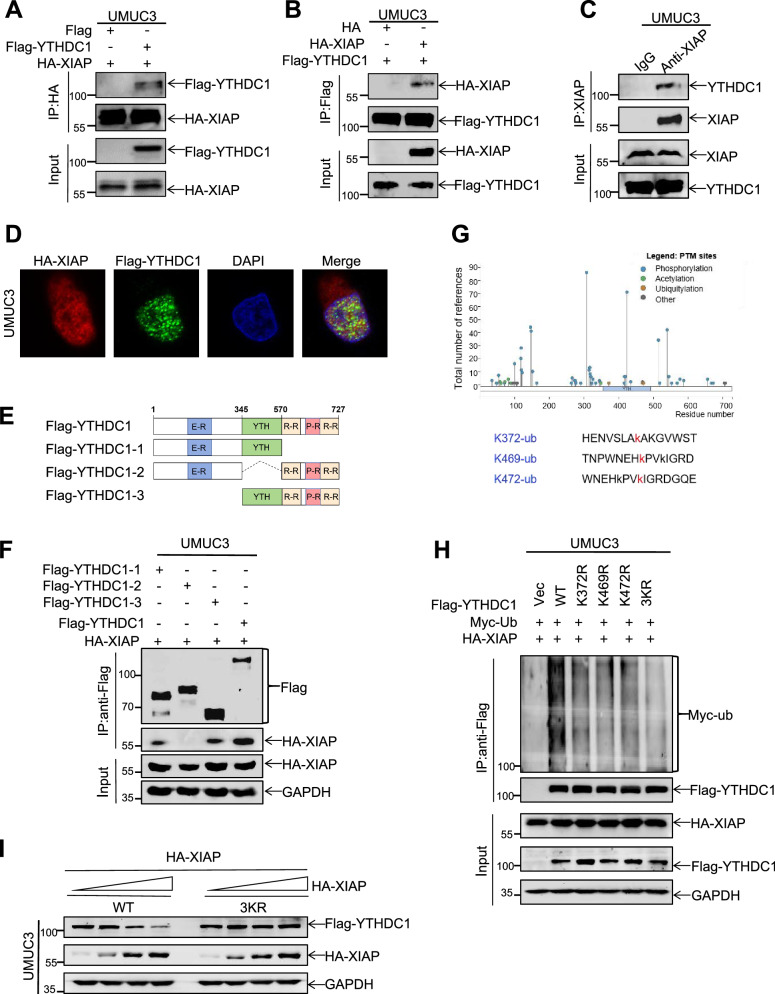
XIAP is a novel ubiquitin E3 ligase for YTHDC1. HA-XIAP and Flag-YTHDC1 were co-transfected into UMUC3 cells. Immunoprecipitation was performed using Anti-HA-tag mAb-Magnetic Beads (**A**) or Anti-DDDDK-tag mAb-Magnetic Beads (**B**) to detect the interaction between XIAP and YTHDC1. **C** UMUC3 cell lysates were subject to immunoprecipitation with control IgG and anti-XIAP antibodies. **D** HA-XIAP and Flag-YTHDC1 were co-transfected and their co-localization was observed by confocal microscopy. **E**, **F** Schematic illustration of YTHDC1 structure (**E**). UMUC3 cells were transfected with the indicated constructs and co-immunoprecipitated with Anti-DDDDK-tag mAb-Magnetic Beads (**F**). **G** YTHDC1 mutant plasmids were constructed at amino acids 372, 469 and 472. **H** The plasmids HA-XIAP, Myc-ub and Flag-YTHDC1 (Vec, WT, K372R, K469R, K472R, 3KR) were transfected into UMUC3 cells and the level of ubiquitination was detected by immunoprecipitation. **I** The plasmids Flag-YTHDC1, Flag-YTHDC1-3KR and HA-XIAP were transfected into the cells, and YTHDC1 degradation rate was detected by western blotting.

Many previous studies have demonstrated that XIAP is an important ubiquitin E3 ligase [34]. Based on our IP-MS data showing that XIAP interacts with YTHDC1, we speculated that XIAP may be a ubiquitin E3 ligase of YTHDC1. To confirm our hypothesis, we co-transfected HA-XIAP and Flag-YTHDC1 into UMUC3 cells, after which protein binding was detected by co-immunoprecipitation assay. As expected, XIAP interacted with YTHDC1 in response to immunoprecipitation using HA or Flag antibody-coated magnetic beads (Fig. 5A, B). Importantly, an interaction between endogenous XIAP and YTHDC1 was detected in UMUC3 cells (Fig. 5C). We also used HA and Flag antibodies to observe localization of the HA-XIAP and Flag-YTHDC1 proteins by immunofluorescence assay and found that these proteins co-localized in UMUC3 cells (Fig. 5D). Next, we detected the location of XIAP and YTHDC1 using the nucleocytoplasmic separation assay (Fig. S4). We then constructed three deletion mutants of YTHDC1 to identify the functional domains of YTHDC1 that mediate the interaction with XIAP (Fig. 5E) [17]. Co-expression of full-length YTHDC1 and the three deletion mutants with XIAP in UMUC3 cells showed that only the YTH domain missing blocked the interaction with XIAP (Fig. 5F). Collectively, these results indicated that XIAP interacts with YTHDC1.

To understand the molecular mechanism via which YTHDC1 is ubiquitinated by XIAP, we searched for specific ubiquitination sites of YTHDC1 in the PhosphoSitePlus database (https://www.phosphosite.org/), including amino sites 372, 469, and 472 (Fig. 5G). Suspecting that these ubiquitination sites may be key to determining the association between XIAP and YTHDC1, we changed Lys to Arg and constructed a series of mutants at three sites separately and in combination. Ubiquitination assays revealed that the mutants both individually and in combination inhibited the ability of XIAP to ubiquitinate YTHDC1 (Fig. 5H). Moreover, the rate of degradation of ectopically expressed YTHDC1_3KR_ was not accelerated, even when the transfection dose of HA-XIAP was increased (Fig. 5I).

### XIAP promotes bladder cancer invasion via the YTHDC1/MMP-2 pathway

**Fig. 6:**
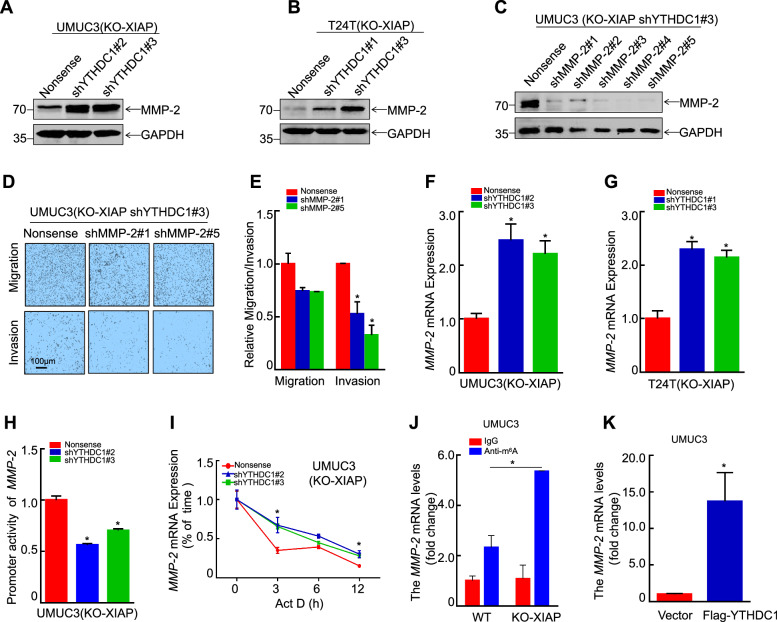
XIAP promotes bladder cancer invasion via the YTHDC1/MMP-2 pathway. **A**, **B** Western blot was used to detect the protein expression levels of MMP-2 in UMUC3/T24T (KO-XIAP shYTHDC1) cells. **C** MMP-2 knockdown efficiency were detected in UMUC3 (KO-XIAP shYTHDC1#3) by western blotting. **D** Transwell assay was used to detect the effects of MMP-2 knockdown on migration and invasion ability of UMUC3 (KO-XIAP shYTHDC1#3). **E** The statistical results of migration and invasion ability of MMP-2 knockdown in UMUC3 (KO-XIAP shYTHDC1#3) cells. **F**, **G** The mRNA levels of MMP-2 were analyzed in UMUC3 (KO-XIAP) (**F**) and T24T (KO-XIAP) (**G**) cells with or without YTHDC1 knockdown. **H** MMP-2 promoter and TK plasmids were co-transfected into UMUC3 (KO-XIAP) cells with or without YTHDC1 knockdown and luciferase activity was measured. **I** MMP-2 mRNA stability assay, in UMUC3 (KO-XIAP) cells with or without YTHDC1 knockdown. **J** MeRIP-qPCR analysis of m^6^A-modified MMP-2 mRNA. **K** RIP assay detected YTHDC1 binding MMP-2 mRNA.

MMPs are proteolytic enzymes that degrade various protein components in the extracellular matrix, destroy the histological barrier to invasion of tumor cells, and play a key role in tumor metastasis [35]. We have published studies demonstrating that XIAP promotes metastasis of bladder cancer by regulating MMP-2 mRNA [36, 37]. Considering that modification of m^6^A can determine mRNA expression levels, we explored whether YTHDC1 was involved in this pathway and found that knockdown of YTHDC1 increased MMP-2 expression in UMUC3/T24T (KO-XIAP) and wild-type cells (Figs. 6A, B and S5). We then knocked down MMP-2 in UMUC3/T24T (KO-XIAP shYTHDC1#3) and control cells to construct stable cell lines (Figs. 6C and S6A, 7A) and tested the invasive ability of indicator cells by transwell assay. We found that knockdown of MMP-2 inhibited the ability of bladder cancer cells to invade in vitro (Figs. 6D, E, S6B, C and S7B, C). These results suggested that MMP-2 is a target of the XIAP-YTHDC1 axis in metastasis of bladder cancer.

**Fig. 7:**
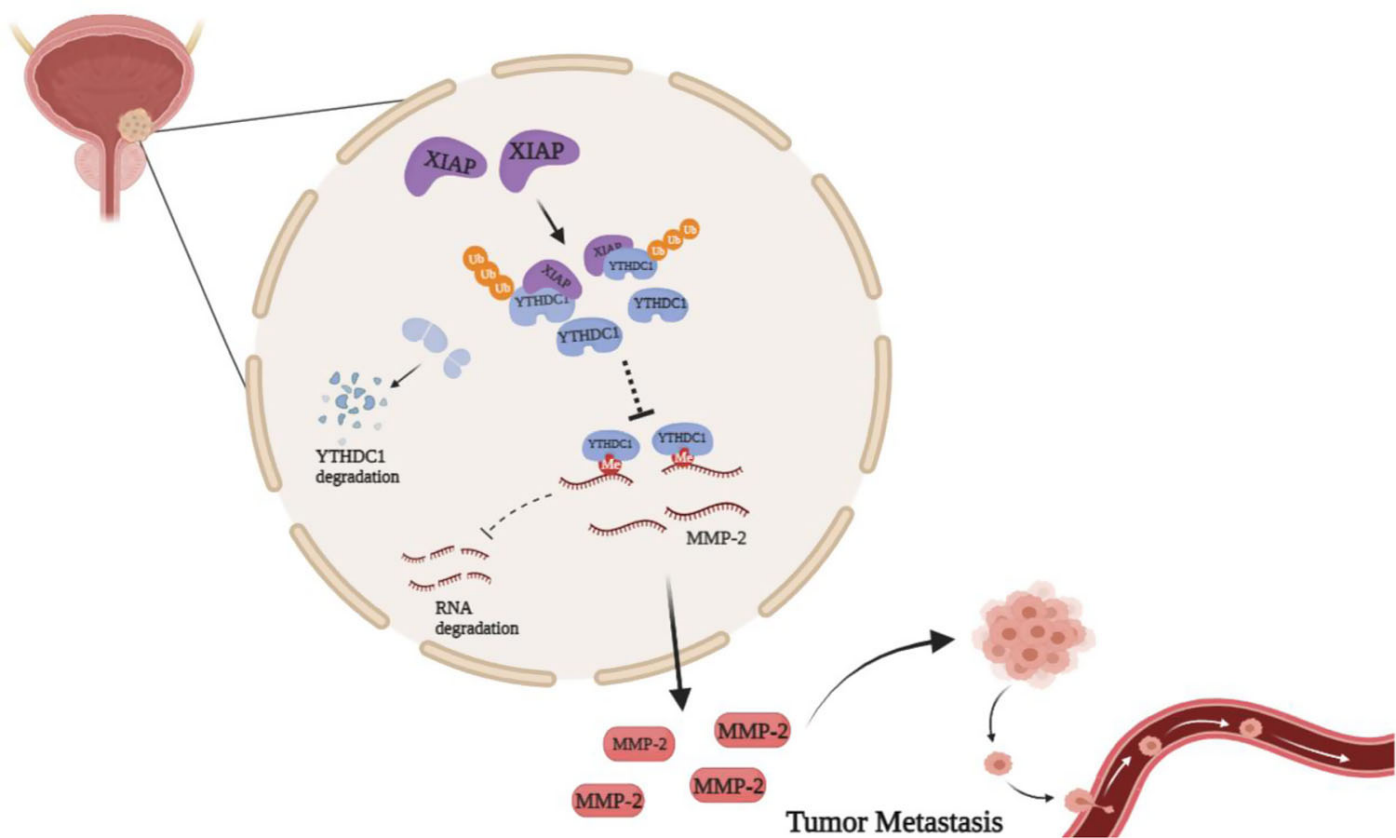
Schematic model of the XIAP-YTHDC1-MMP-2 axis in bladder cancer metastasis. XIAP promotes the ubiquitination and degradation of YTHDC1, and up-regulates the expression of MMP-2 to promote the metastasis of bladder cancer cells. This study provides potential molecular targets for bladder cancer treatment.

Next, we assessed the abundance of MMP-2 mRNA in the indicated cells to examine how XIAP regulates expression of MMP-2 through YTHDC1. Real-time PCR showed that knockdown of YTHDC1 led to a marked increase in abundance of MMP-2 mRNA in UMUC3/T24T (KO-XIAP) cells, suggesting that expression of MMP-2 protein was correlated with transcript levels (Fig. 6F, G). To examine this possibility, we performed a pGL3-MMP-2 promoter-luciferase reporter assay and found that knockdown of YTHDC1 resulted in inconsistent changes in MMP-2 promoter activity in UMUC3 (KO-XIAP) cells (Fig. 6H). Considering that YTHDC1 can regulate gene expression by affecting the stability of mRNA [38], we tested the stability of MMP-2 by adding actinomycin D (20 μg/mL) to inhibit new mRNA transcription. We found that knockdown of YTHDC1 slowed the degradation of MMP-2 mRNA in UMUC3 (KO-XIAP) cells (Fig. 6I). Furthermore, methylated RNA immunoprecipitation (MeRIP)-qPCR showed that m^6^A-modified MMP-2 mRNA levels were significantly increased in UMUC3 (KO-XIAP) cells (Fig. 6J). The RIP assay also showed that YTHDC1 pulled down MMP-2 mRNA (Fig. 6K). We confirmed that XIAP regulated the MMP-2 mRNA level via YTHDC1 by affecting the stability of mRNA. These findings clarified the molecular mechanism via which the XIAP/YTHDC1/MMP-2 axis promotes metastasis of bladder cancer (Fig. 7).

## Discussion

The biological effects of XIAP are widely attributed to apoptosis, autophagy, and signaling pathways, and it is known that XIAP is an important ubiquitin E3 ligase [39]. In this study, we demonstrated that XIAP is also a ubiquitin E3 ligase of YTHDC1, accelerating the degradation of YTHDC1 and promoting metastasis of bladder cancer.

XIAP is overexpressed in a variety of malignancies, including liver, bladder, and breast cancers, and promotes proliferation and metastasis of cancer cells and drug resistance [40, 41]. As a ubiquitin E3 ligase, XIAP exerts its oncogenic effect by ubiquitinating a variety of substrates. Previous studies have suggested that XIAP degradation MDM2 negatively regulates the expression of p53, mediates inhibition of autophagy, and ultimately promotes tumorigenesis [9]. XIAP ubiquitinates RIPK2 and recruits neutrophils to inhibit growth of melanoma [42]. However, although XIAP is known to promote ubiquitination of various substrates, many of the substrates are currently unknown. Therefore, we investigated the direct substrates of XIAP and unexpectedly found that XIAP binds to and ubiquitinates YTHDC1 and that expression of YTHDC1 is reversed when XIAP loses ubiquitination activity, suggesting that XIAP may be the ubiquitin E3 ligase of YTHDC1. We are planning further investigation of the XIAP domain and its interactions with YTHDC1, which may be key in the treatment of bladder cancer.

N6-methyladenosine is a widespread RNA modification that dynamically and reversibly regulates the translation, cleavage, transport, and processing of modified RNA. Abnormal modification of m^6^A can affect gene expression and induce various types of cancer, including acute myeloid leukemia, glioblastoma multiforme, and lung cancer [43–45]. There has been some research on the function of METTL3 and METTL14 in bladder cancer. METTL3 promotes tumor proliferation and PD-L1-mediated immune escape while METTL14 can inhibit tumorigenesis in the bladder [46–48]. Researchers are becoming increasingly aware of the effects of the YTH family in bladder cancer. YTHDF2 promotes progression of bladder cancer, whereas YTHDC1 can increase drug sensitivity and inhibit progression of malignancy [25, 49]. The above-mentioned studies suggest that regulating modification of m^6^A is crucial in the treatment of bladder cancer.

YTHDC1, a nuclear m^6^A reader, recognizes the m^6^A of RNA and controls its function and fate [22, 26, 50]. YTHDC1 realized oncogenic RNA splicing of tumor suppressor RBM4 during progression of cancer and was found to control the DNA replication regulator MCM4, which promotes proliferation and survival of acute myeloid leukemia cells [24, 51]. Many studies have suggested that YTHDC1 mainly regulates the RNA levels of downstream molecules. However, the mechanism that controls expression of YTHDC1 has rarely been investigated. Our finding that XIAP, a ubiquitin E3 ligase of YTHDC1, regulates expression of YTHDC1 via the proteasome pathway has important implications. Knockout or knockdown of XIAP reduced the ubiquitination level and degradation of YTHDC1. We hypothesize that YTHDC1 may be regulated by other ubiquitin E3 ligases, identification of which may provide another novel strategy for the treatment of bladder cancer.

MMP-2 degrades the basement membrane and extracellular matrix and plays an important role in tumor invasion [52]. MMP-2 is overexpressed in breast, gastric, and liver cancers and promotes metastasis of tumor cells [53]. In a previous study, we found that MMP-2 is an important molecular marker of metastasis of bladder cancer [12, 54]. MMP-2 has been found to affect mRNA levels as a result of differential expression of transcription factors, but modification of RNA has not been reported before. The findings of our present study suggest that XIAP and YTHDC1 interact to regulate the stability of MMP-2 mRNA and promote metastasis of bladder cancer. This study is the first to demonstrate that XIAP is involved in regulation of m^6^A modification, which should lead to new avenues of research in bladder cancer.

Overall, this study provides new insights into the mechanism via which XIAP regulates metastasis of bladder cancer, demonstrating in particular that XIAP acts as a novel E3 ligase for YTHDC1. Our functional studies have shown that XIAP upregulated expression of MMP-2 by downregulating YTHDC1 and promoting metastasis of bladder cancer cells. These findings demonstrate that XIAP is an important regulator of YTHDC1 and that the XIAP/YTHDC1/MMP-2 axis plays an important role in bladder cancer.

## Materials and methods

### Plasmids, reagents, and antibodies

The XIAP knockout CRISPR/Cas9 system was constructed using PX458M vector. Short hairpin RNA (shRNA) specific targeting human XIAP and MMP-2 were purchased from Open Biosystems (Huntsville, AL, USA). YTHDC1-shRNA and control plasmids were purchased from the Public Protein/Plasmid Library. Overexpression XIAP, XIAP-ΔRING, XIAP_H467A_ and XIAP_F495A_ were constructed into pEBB plasmid. YTHDC1, YTHDC1-1 (aa1-570), YTHDC1-2 (aa1-345, 570-727), YTHDC1-3 (aa346-727) were constructed into pEnCMV-3×Flag plasmids, respectively. Construction of MMP-2 double luciferase reporter plasmid in pGL3-Basic with KpnI and HindIII restriction enzymes. MG132 was obtained from Selleck Chemicals (Houston, TX, USA). Cycloheximide was purchased from Calbiochem (San Diego, CA, USA). The antibodies used as followed: anti-XIAP (610763, BD Biosciences), anti-MMP-2 (sc-13594, Santa Cruz), anti-HA (3724S, CST), anti-Flag (14793S, CST), anti-Myc (2276S, CST), anti-YTHDC1 (14392-I-AP, Proteintech), and anti-GAPDH (10494-1-AP, Proteintech).

### Cell culture and transfection

Human BC cell lines UMUC3 and T24T were cultured at 37 °C in a 5% CO_2_ incubator, as previously described [55, 56]. UMUC3 cells were cultured in DMEM medium supplemented with 10% FBS (GIBCO). T24T cells were cultured in DMEM/F12 medium supplemented with 5% FBS. Plasmids were transfected into cells using PolyJet^TM^ transfection reagent (SignaGen Laboratories, Rockville, MD, USA). Stable cell lines were selected using flow cytometry or antibiotics.

### SDS–PAGE and western blotting

Cell samples were lysed using boiling buffer (1 M Tris-HCl, PH = 7.4, 10% SDS, 100 mM Na3VO4). Protein sample concentration is determined using NanoDrop 2000 (Thermo Fisher Scientific, USA). Equal amounts of protein were separated by PAGE Gel Fast Preparation Kit (EpiZyme Biotechnology, China). Samples in SDS-PAGE were transferred to PVDF membranes using eBlot^TM^ L1 (Genscript Biotech Corporation). PVDF was blocked with 5% milk, and incubated with primary antibody for 12 h at 4 °C, and incubated with secondary antibodies for 2 h and finally exposed to ECF (RPN5785, GE Healthcare) using Typhoon FLA 7000 (GE Healthcare).

### Real-time PCR

Cells were lysed using TRIzol (Invitrogen, USA), and total RNA was extracted according to the manufacturer’s protocol. RNA was reverse-transcribed to cDNA using the PrimeScript RT reagent Kit (RR037A, Takara). mRNA expression was measured using Taq Pro Universal SYBR qPCR Master Mix (Q712-02, Vazyme) in QuantStudio™ 6 Flex real-time fluorescent quantitative PCR system (Thermo Fisher). Amplification of genes using specific primers is as follows: human *YTHDC1* (forward: 5’-CTG GTT TGA TCT TTT CGG ACA G-3’, reverse: 5’-AGT GAC TCT GGT TCT GAA TCT G-3’), human *MMP-2* (forward: 5’-TTC CGC TTC CAG GGC ACA-3’, reverse: 5’-CAC CTT CTG AGT TCC CAC CAA-3’), and human *GAPDH* (forward: 5’-ATC AAT GGA AAT CCC ATC ACC A-3’, reverse: 5’-GAC TCC ACG ACG TAC TCA GCG-3’).

### Immunohistochemistry

The pathological sections were baked at 65 °C and then dewaxed using xylen and tissue hydration was carried out in different concentrations of alcohol. The Citrate Antigen Retrieval Solution (pH = 6.0 0.01 M) repairs antigens by microwave heating. Endogenous peroxidase was blocked with 3% H_2_O_2_, followed by blocking nonspecific sites with 5% BSA. The primary antibody was incubated at 4 °C overnight, then the secondary antibody and SABC at 37 °C. The chromogenic reaction was performed using DAB (59718, Abcam) followed by counterstained with hematoxylin. Images were acquired and the integrated optical density (IOD) of each stained area (IOD/area) was analyzed.

### Immunoprecipitation

Cells are washed with cold PBS and lysed in cell lysis buffer (9803, CST) containing complete protein inhibitor cocktail (04693116001, Roche). The cell lysates were centrifuged 14,000 × *g* for 10 min at 4 °C. The protein concentration in the whole cell lysate was detected by BCA (23227, Thermo Fisher) and the samples were divided into Input and IP. The IP samples were incubated with the indicated XIAP antibodies (sc-55550, Santa Cruz) and protein A/G agarose beads (Santa Cruz) at 4°C for immunoprecipitation. Similarly, IP samples were incubated with tag magnetic beads (anti-HA-tag: M180-11; Anti-DDDDK-tag: M185-11R, MBL) were incubated at 4 °C for co-immunoprecipitation. Protein complexes were resuspended with boiling buffer, and detected by western blotting.

### RIP-qPCR and MeRIP-qPCR

UMUC3 cells were cultured in 10-cm dishes to 70–80% confluence and then transfected with Flag-YTHDC1 or vector control for 24 h. RIP analysis was performed using YTHDC1 (14392-1-AP) and RNA immunoprecipitation kit (Bes5101, BersinBio). UMUC3 (KO-XIAP) and wild-type cells were cultured in 10-cm dishes to 90% confluence, after which MeRIP analysis was performed using an m^6^A MeRIP Kit (Bes5203, BersinBio). Real-time PCR was used to measure the mRNA content in immune complexes.

### Immunofluorescence microscopy

Cells were attached to a circle microscope cover glass, cleaned with PBS, and fixed with 4% PFA. 0.3% Triton X-100 (P0096, Beyotime Biotechnology) permeable cells for 20 min, rapid blocking solution (P0220, Beyotime Biotechnology) blocking cells for 10 min. Primary antibody was incubated overnight at 4 °C and fluorescent antibody was incubated at 37 °C for 1 h. Cells were counterstained using DAPI-containing anti-fluorescent quencher (P0131, Beyotime Biotechnology) and transferred to glass slides. Images were captured by Confocal laser scanning microscopy.

### Nucleocytoplasmic separation assay

NE-PER (R) Nuclear and Cytoplasmic Extraction Reagents (78833, Thermo Fisher) were used to perform nucleocytoplasmic separation assay. Maintain the volume ratio of CER I: CER II: NER reagents at 200:11:100 µL, respectively. Briefly, 2 × 10^6^ UMUC3 cells were harvested by trypsin-EDTA, added ice-cold CER I and incubated for 10 min. CER II was added to the tubes, incubated for 1 min, and the cytoplasm was collected by centrifugation at 16,000 *× g* and 4 °C. The insoluble fraction was suspended with ice-cold NER and vortexed every 10 min, for a total of 40 min. Nuclear extracts were separated by centrifugation and detected by western blotting.

### Transwell assay

Transwell assays were performed using Corning chambers (353097, Corning) according to the manufacturer’s instructions. Matrigel (354234, Corning) tiled at the bottom as an invasion chamber. 3 × 10^4^ T24T and 4 ×10^4^ UMUC3 in serum-starved medium were added to the upper layer, and added complete medium (10% FBS) to the lower chamber. After the cells were cultured in 5% CO_2_ at 37 °C for 24 h, fixed with 4% PFA, permeabilized with methanol for 30 min, and stained with Giemsa stain (48900, Sigma-Aldrich) for 30 min. Cells in the upper chamber were wiped, and five different images were randomly captured and counted.

### Dual-luciferase reporter assay

pRL-TK plasmid and MMP-2 promoter plasmid were 1:10 and co-transfected into UMUC3 and T24T cells for 24 h. Luciferase activity was measured using Dual-Glo Luciferase Assay Kits (E1960, Promega). Add 1 ×lysis Buffer shock cells for 15 min, transfer 40 μL to a black 96-well plate, and mix with Luciferase Assay Reagent II to detect fluorescence values. Add 1 ×Stop & Glo to detect TK values and save on the Centro LB 960 luminometer (cr-artisan, China).

### Mouse tumor models

Female nude mice were purchased from GemPharmatech (Nanjing, Jiangsu, China) and raised in the SPF-level experimental animal facility of Wenzhou Medical University. Nude mice are randomly grouped, then injected with 100 μL of 3 × 10^6^ cells of T24T cells. Feeding until about 12 weeks, lung tissue was taken to count the number of tumors, photographed with a stereomicroscope (ZEISS), and fixed with Bouin’s solution. The tissue was paraffin-embedded and sectioned, stained with HE (G1120, Solarbio), and photographed.

### Clinical specimens

Experiments involving human subjects were collected from The First Affiliated Hospital of Wenzhou Medical University (Wenzhou, Zhejiang, China) and signed the informed consent with all patients before the research started. BC tissues and adjacent normal tissues were collected from 17 patients shown in Supplementary Table 1.

### Statistical analysis

The Student’s *t*-test was used to determine significant differences, and *p* < 0.05 was considered as a significant difference between compared groups.

## Supplementary information

Supplementary Material

Original western blots

## Data Availability

The data that support the findings of this study are openly available in ProteomeXchange. Proteomics data accession number is PXD055868.
