# Supplementary material for: XIAP promotes metastasis of bladder cancer cells by ubiquitylating YTHDC1
Source: Cell Death Dis. 2025 Mar 25;16(1):205. doi: 10.1038/s41419-025-07545-9 (PMC11937301; doi:10.1038/s41419-025-07545-9)
Supplement: Supplementary file 1 — Supplementary Material [file 41419_2025_7545_MOESM1_ESM.pdf]

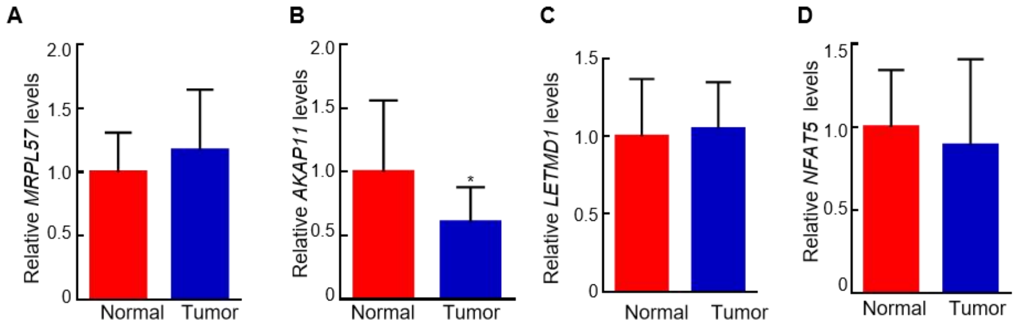

Figure S1. The expressions of MRPL57 **(A)**, AKAP11 **(B)**, LETMD1 **(C)**, and NFAT5 **(D)** in TCGA database.

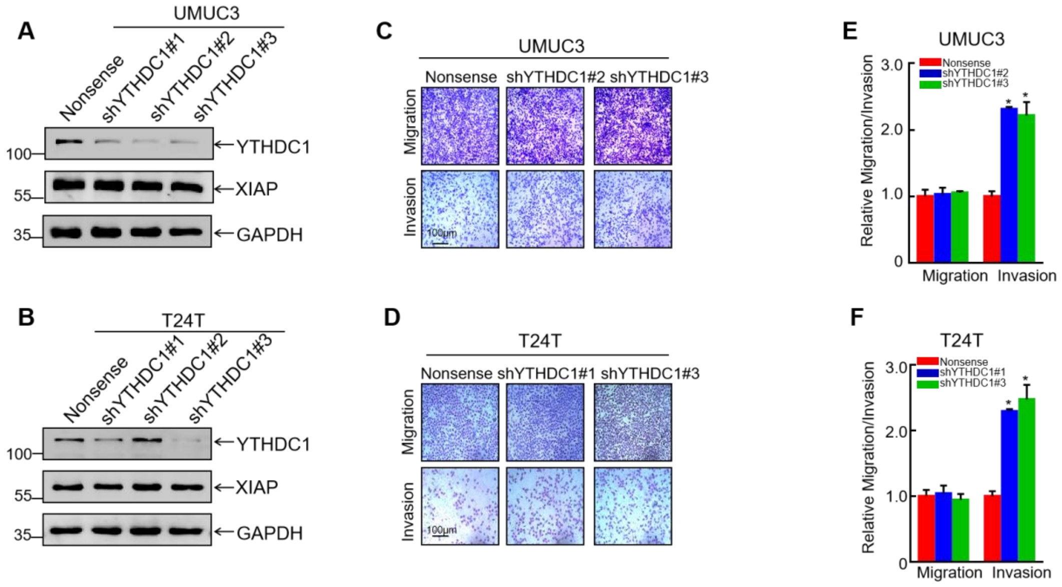

Figure S2. A, **B** Plasmids nonsense, shYTHDC1#1, shYTHDC1#2 and shYTHDC1#3 are packed with lentvirus to infect UMUC3 **(A)** and T24T cells **(B)**, and the knockdown efficiency is detected by Western blotting. **C**, **D** Transwell assay was used to detect the effects of YTHDC1 knockdown on migration and invasion ability of UMUC3 **(C)** and T24T cells **(D)**. **E**, **F** The statistical results of migration and invasion ability of YTHDC1 knockdown in UMUC3 **(E)** and T24T cells **(F),** $*p<0.05$.

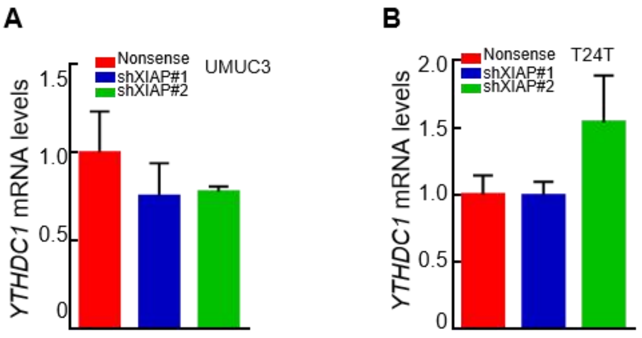

Figure S3. YTHDC1 mRNA levels were detected in XIAP knockdown UMUC3 **(A)** and T24T **(B)** cells by real-time PCR.

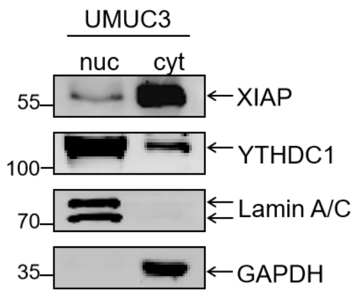

Figure S4. Nucleo-plasmic separation assay was performed in UMUC3 cells to detect XIAP and YTHDC1 localization.

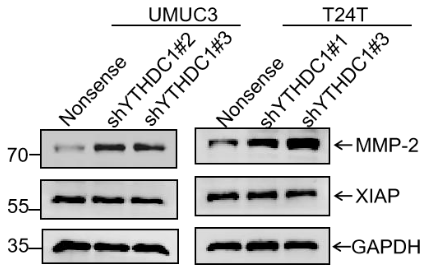

Figure S5. Western blot was used to detect the protein expression levels of MMP-2 in UMUC3/T24T (shYTHDC1) cells.

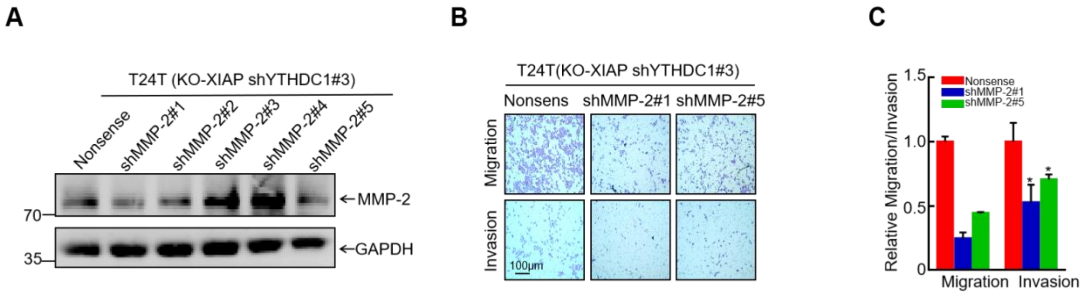

Figure S6. **A** MMP-2 knockdown efficiency were detected in T24T (KO-XIAP shYTHDC1#3) cells by Western blotting. **B** Transwell assay was used to detect the effects of MMP-2 knockdown on migration and invasion ability of T24T (KO-XIAP shYTHDC1#3) cells. **C** The statistical results of migration and invasion ability, *p<0.05.

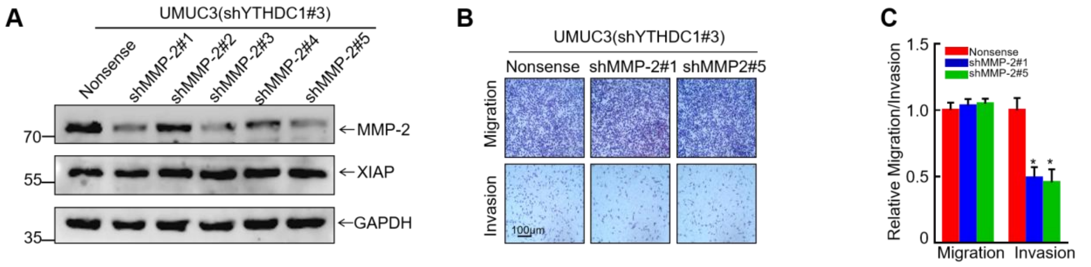

Figure S7. **A** MMP-2 knockdown efficiency were detected in UMUC3 (shYTHDC1#3) cells by Western blotting. **B** Transwell assay was used to detect the effects of MMP-2 knockdown on migration and invasion ability of UMUC3 (shYTHDC1#3) cells. **C** The statistical results of migration and invasion ability, *p<0.05.

Table S1. Clinical case information of 17 patients

case(NO#)	medical record NO	gender	age	NMIBC/MIBC	stage
1#	1791873	male	66	MIBC	T2b
2#	1919124	male	64	MIBC	T2b
3#	1941718	male	70	MIBC	T2a
4#	2211748	male	55	NMIBC	T1
5#	3019780	male	52	MIBC	T2a
6#	3178143	male	79	MIBC	T2b
7#	3185908	male	68	NMIBC	Ta
8#	3190758	male	78	MIBC	T4a
9#	3191919	male	82	NMIBC	Ta
10#	3192384	male	73	NMIBC	T1
11#	3192465	male	62	MIBC	T3
12#	3197219	male	65	NMIBC	T1
13#	3199069	male	67	MIBC	T3a
14#	3203757	male	68	NMIBC	T1
15#	3204805	male	78	NMIBC	T1
16#	3209293	male	71	NMIBC	T1
17#	9242379	male	55	MIBC	T2b
