# Supplementary material for: XIAP promotes metastasis of bladder cancer cells by ubiquitylating YTHDC1
Source: Cell Death Dis. 2025 Mar 25;16(1):205. doi: 10.1038/s41419-025-07545-9 (PMC11937301; doi:10.1038/s41419-025-07545-9)

Figure 1A

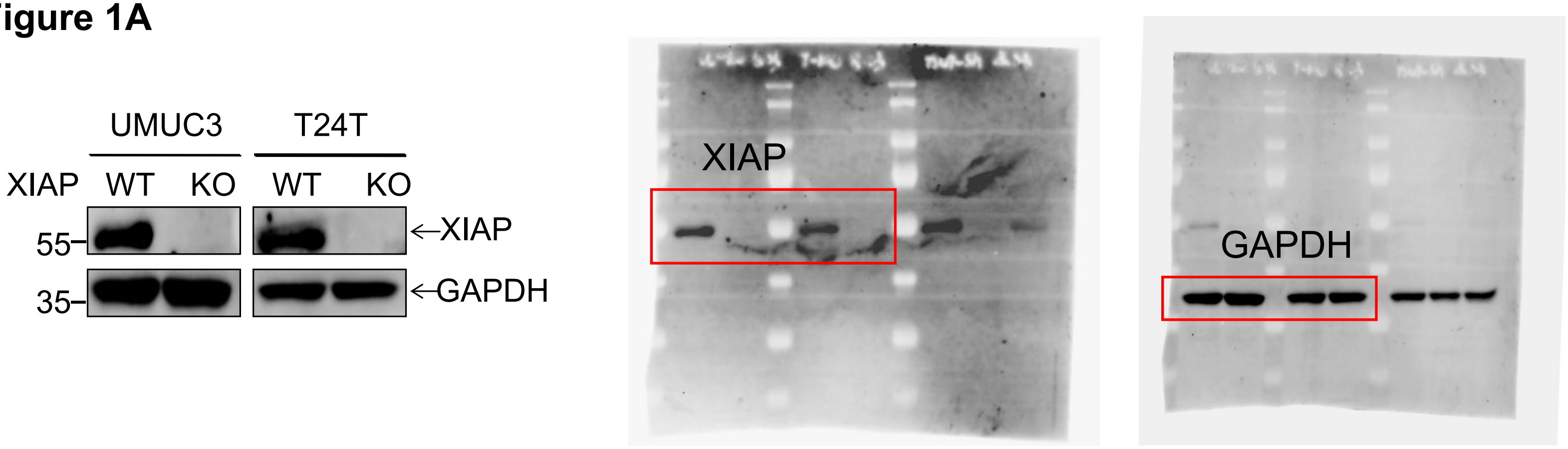

Figure 1B

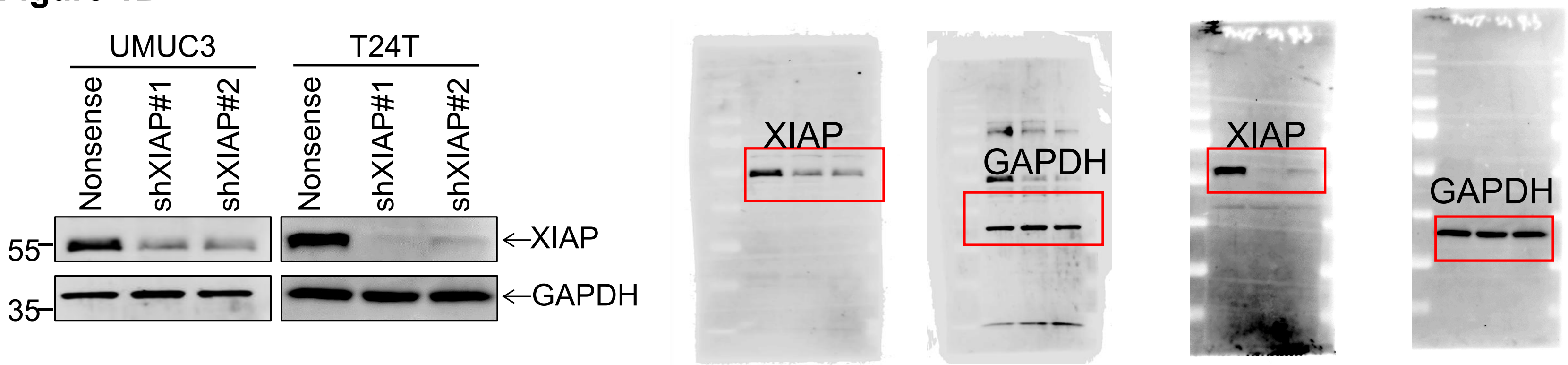

Figure 1C

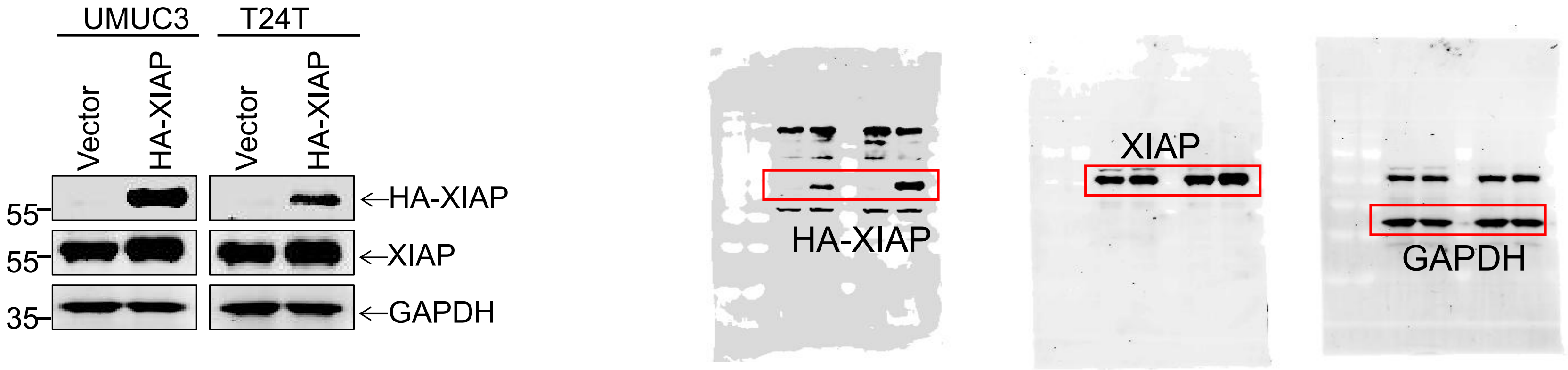

Figure 2H

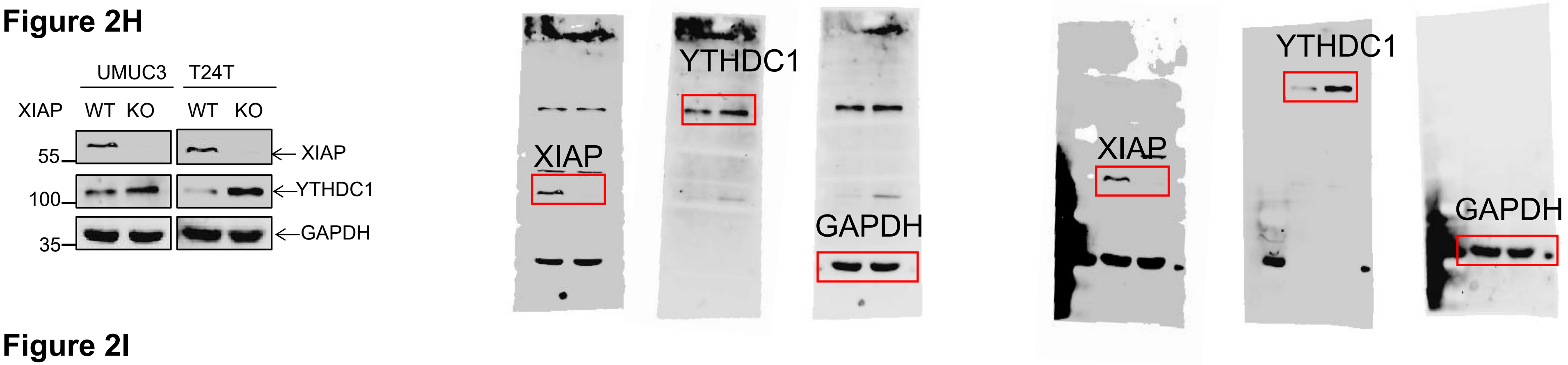

Figure 2I

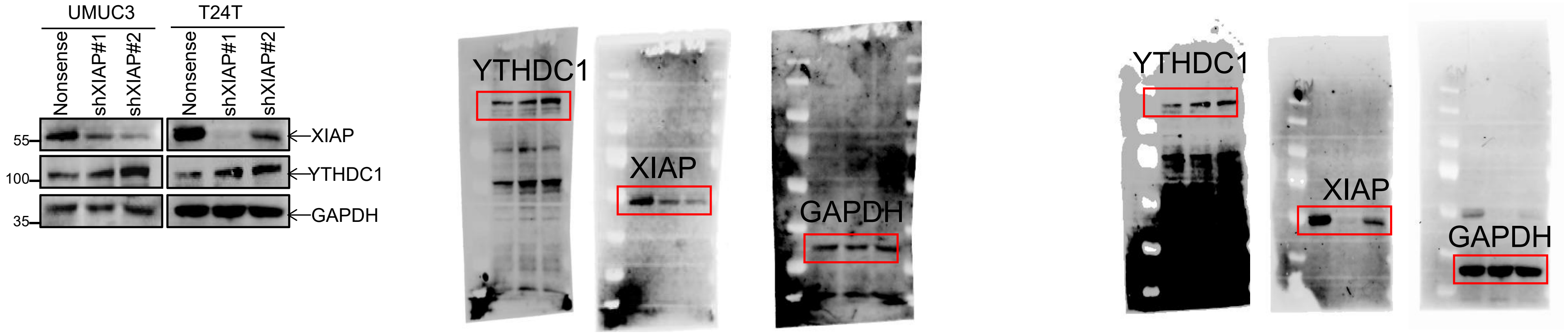

Figure 2J

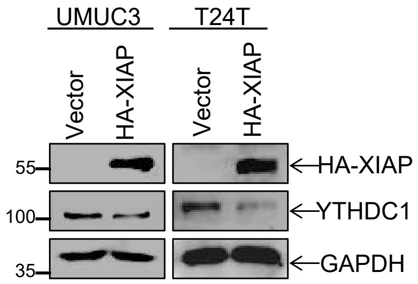

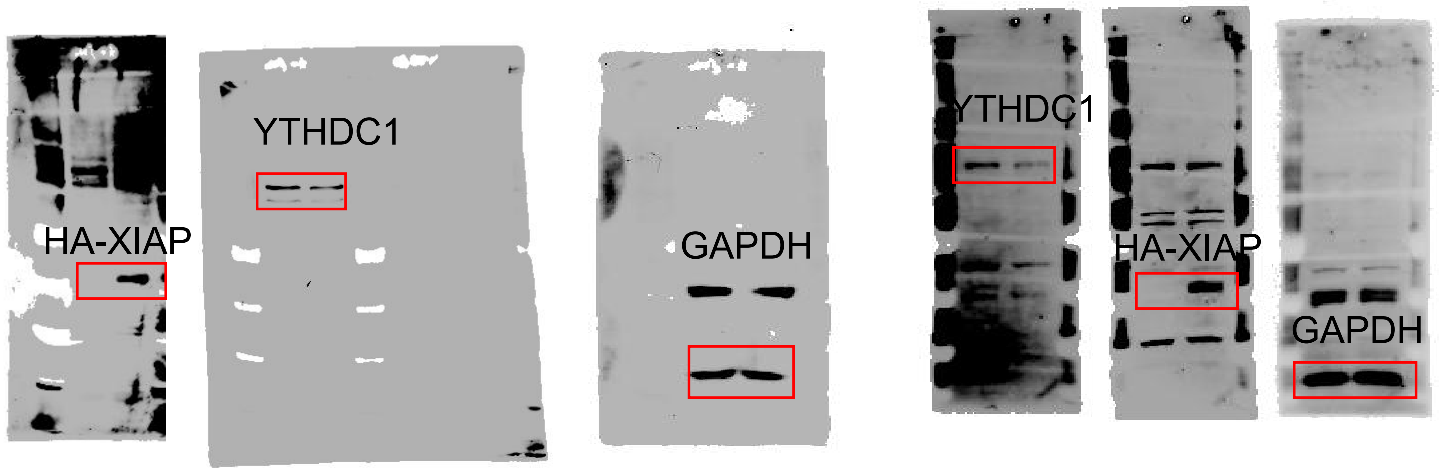

Figure 3A

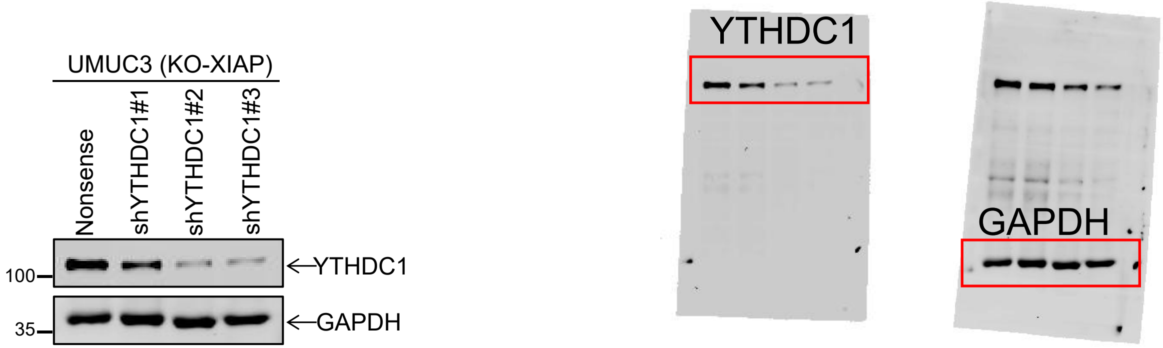

Figure 3D

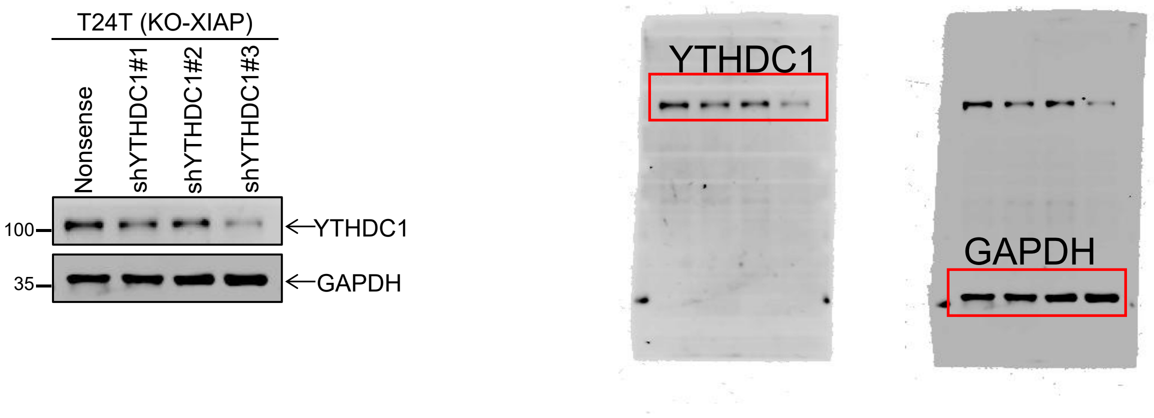

Figure 4A

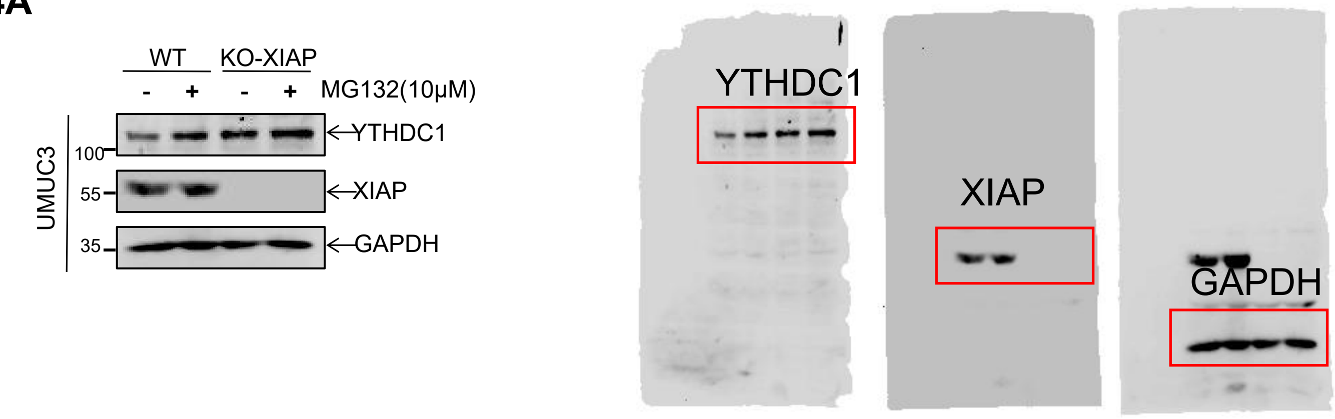

Figure 4B

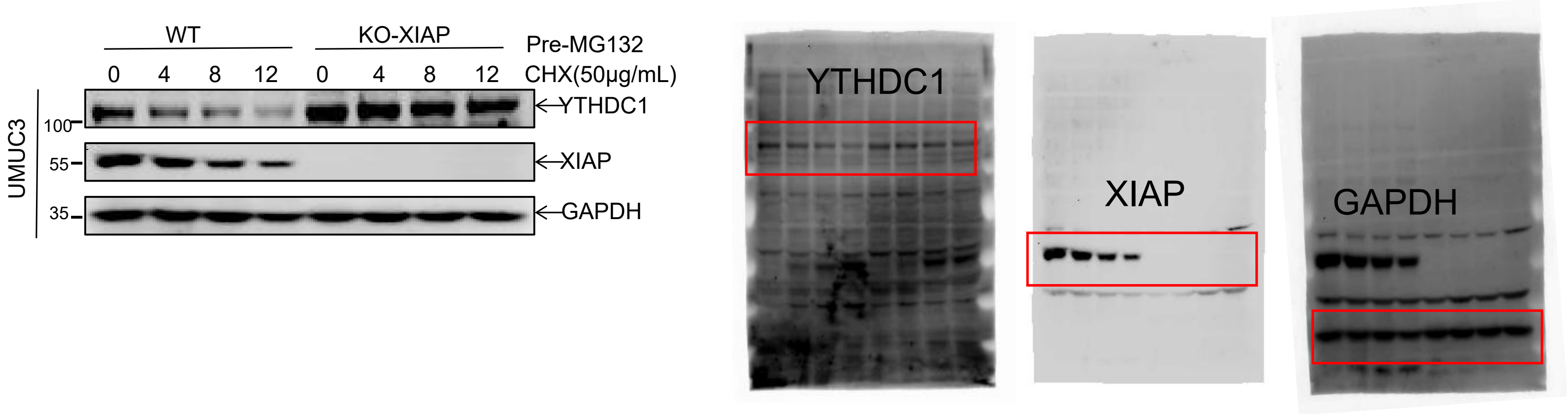

Figure 4C

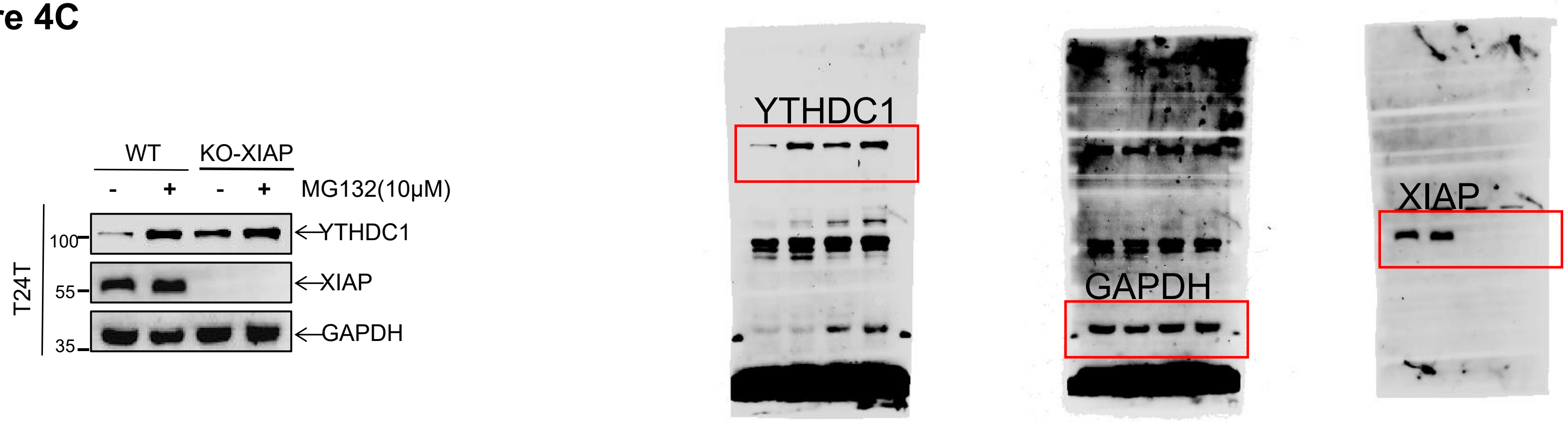

Figure 4D

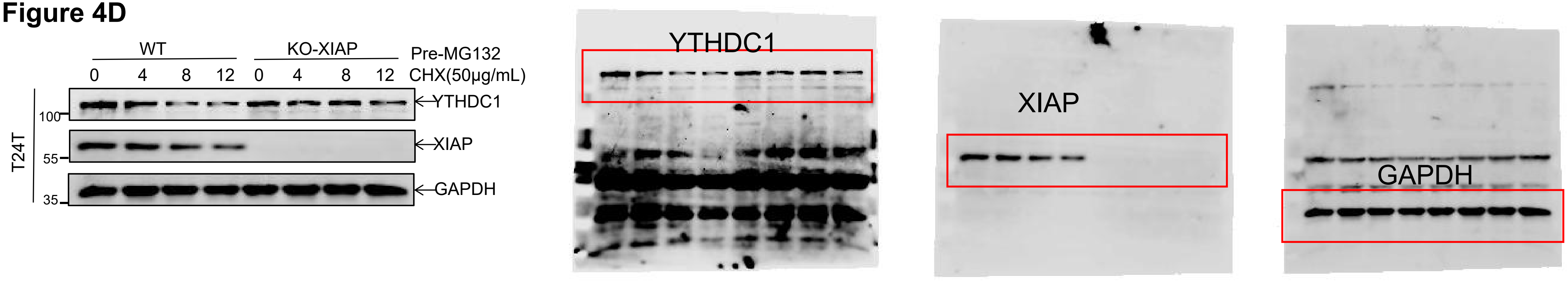

Figure 4E

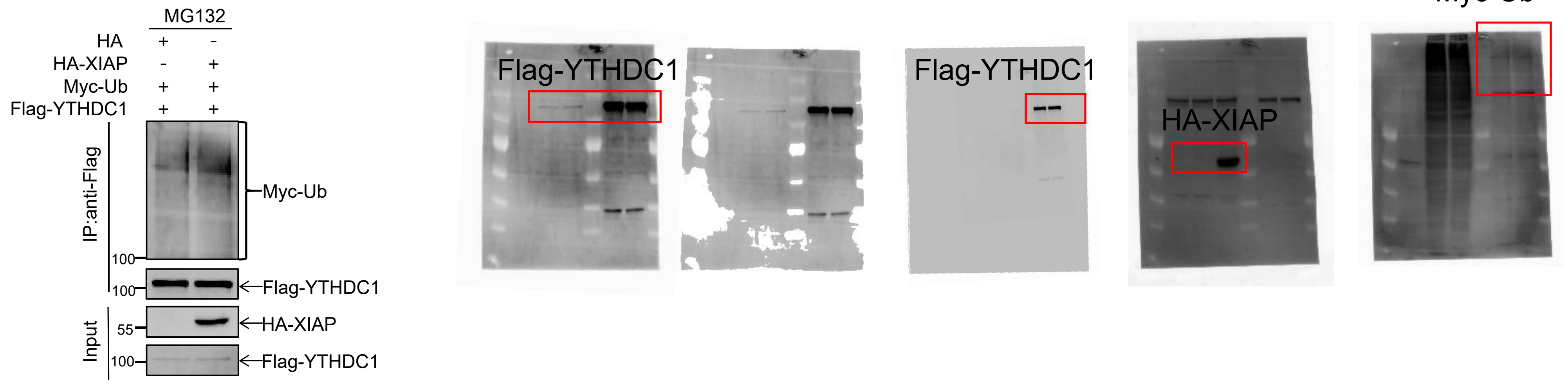

Figure 4F

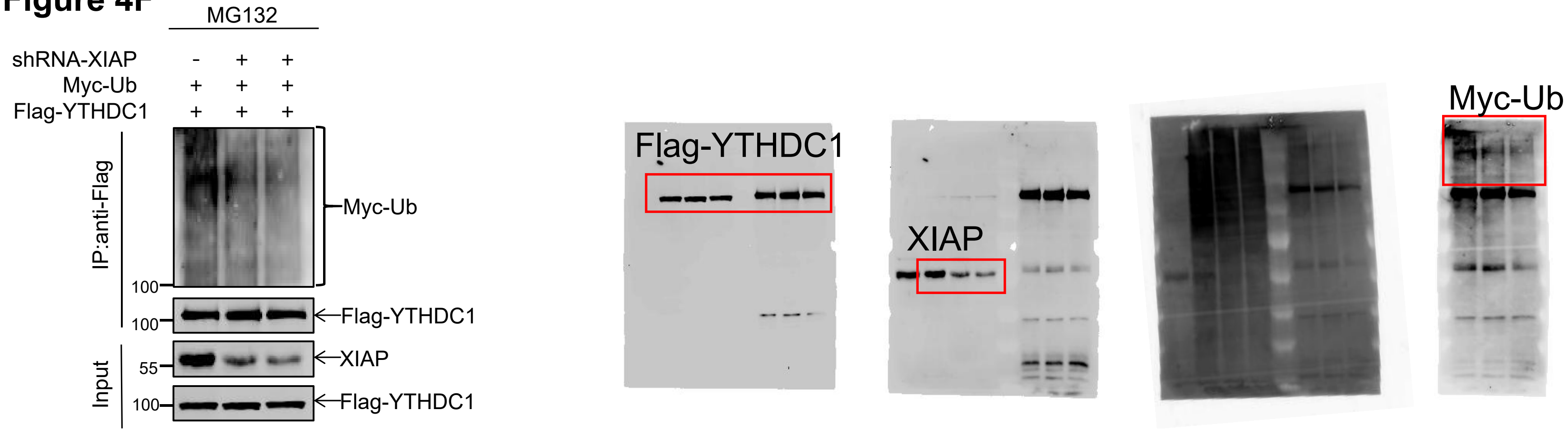

Figure 4G

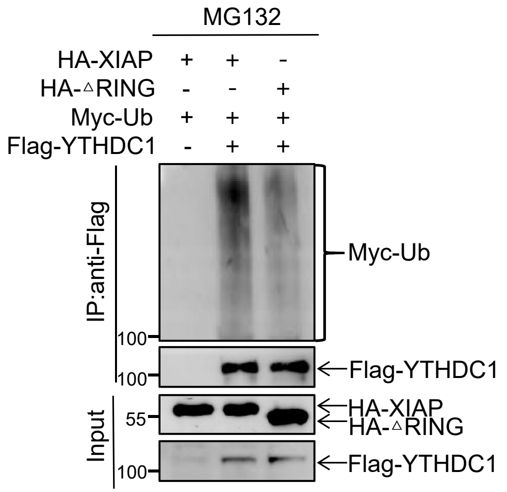

MG132
HA-XIAP + + -
HA-△RING - - +
Myc-Ub + + +
Flag-YTHDC1 - + +
IP:anti-Flag
Input
100
100
55
100
Myc-Ub
Flag-YTHDC1
HA-XIAP
HA-△RING
Flag-YTHDC1

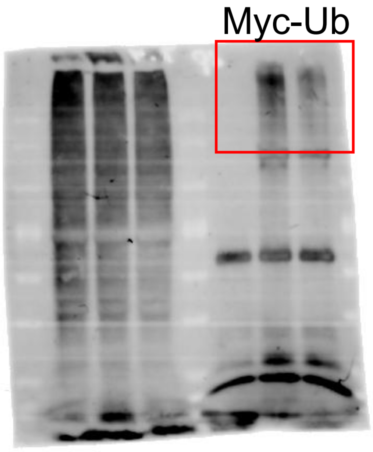

Myc-Ub

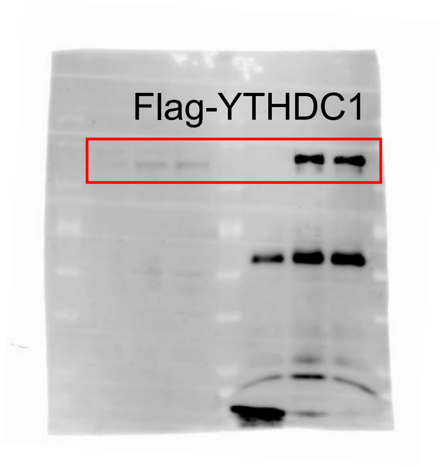

Flag-YTHDC1

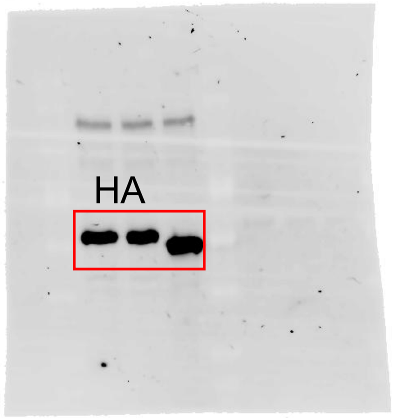

HA

Figure 4H

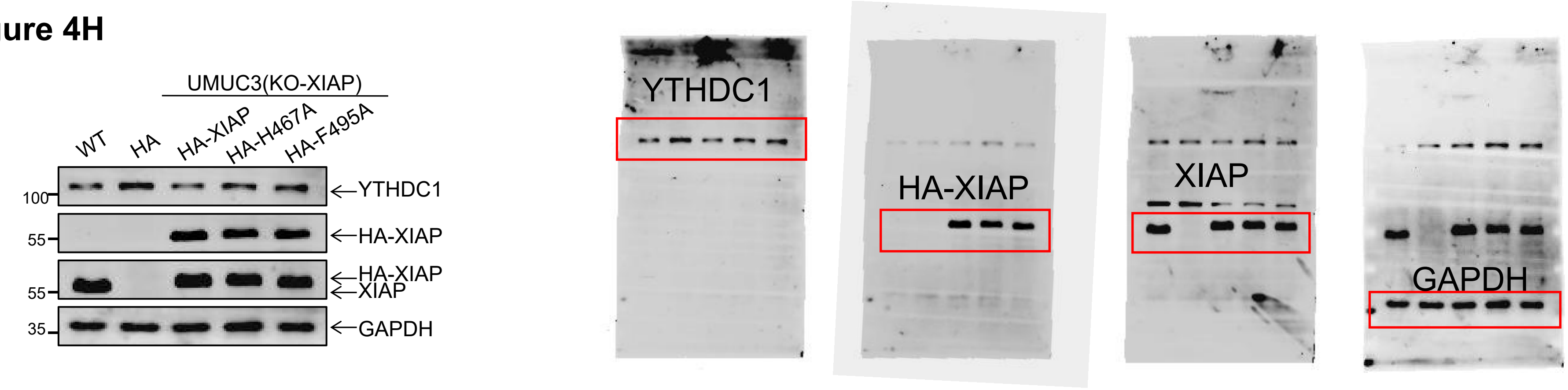

Figure 4I

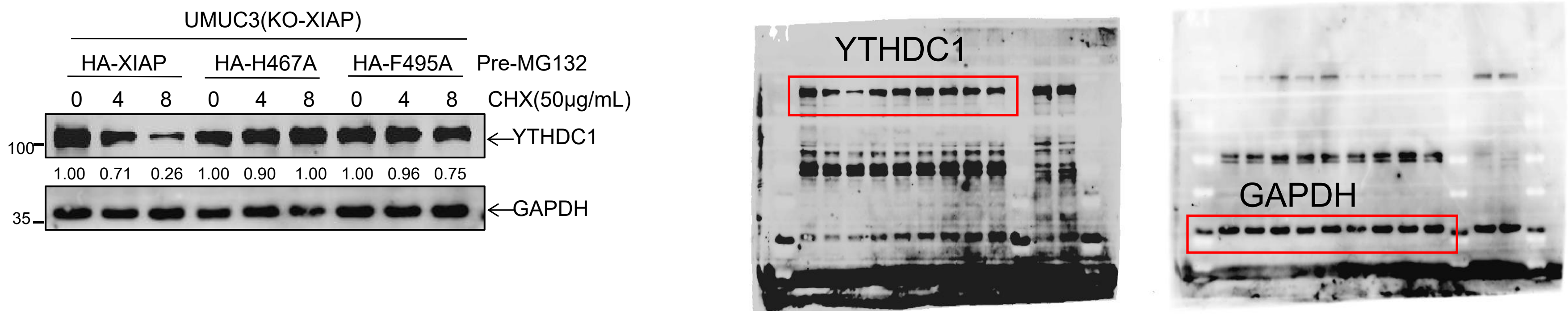

Figure 5A

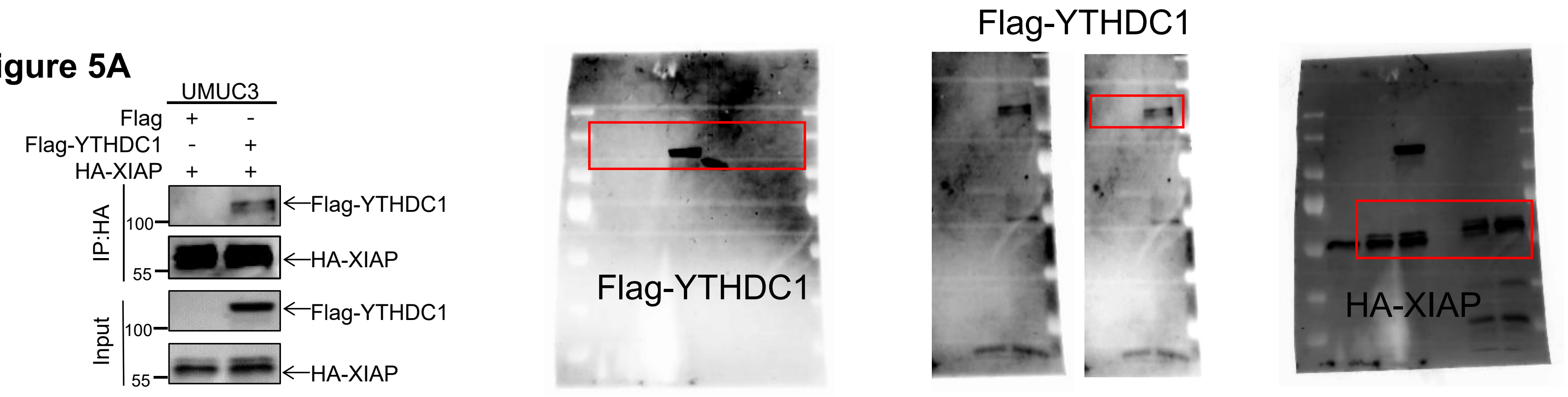

Figure 5B

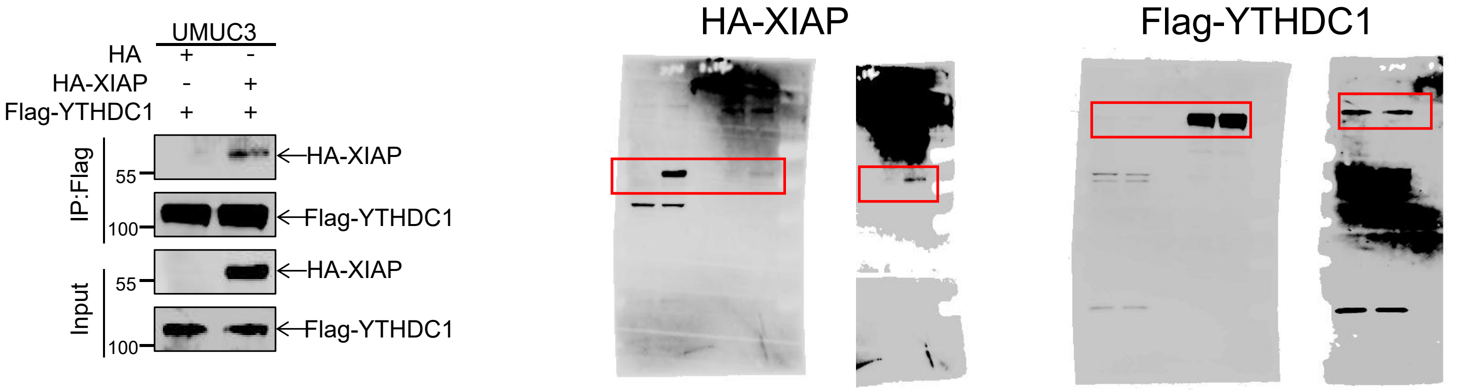

Figure 5C

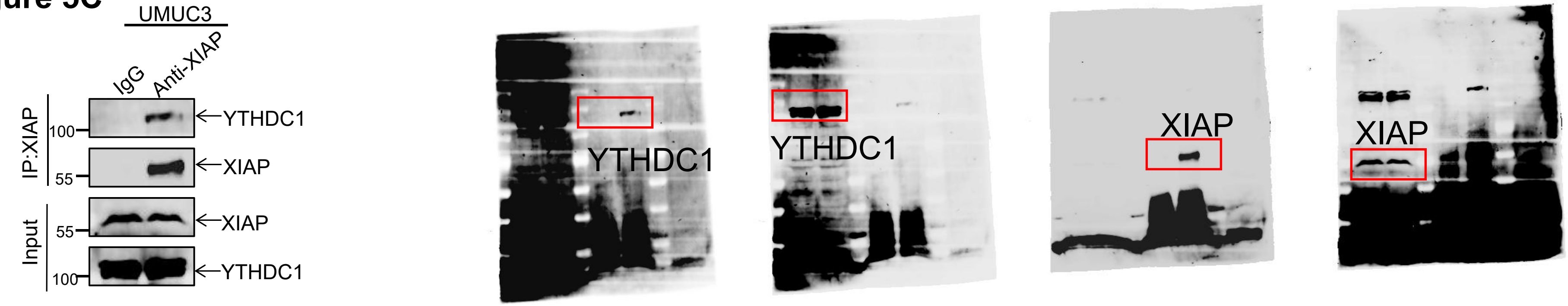

Figure 5F

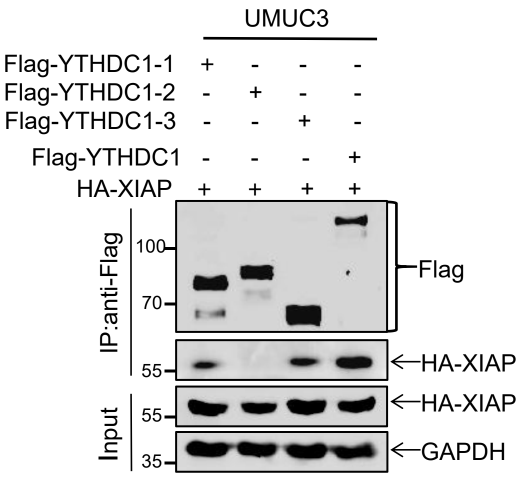

UMUC3
Flag-YTHDC1-1 + - - -
Flag-YTHDC1-2 - + - -
Flag-YTHDC1-3 - - + -
Flag-YTHDC1 - - - +
HA-XIAP + + + +
IP:anti-Flag
100
70
55
Flag
HA-XIAP
Input
55
35
HA-XIAP
GAPDH

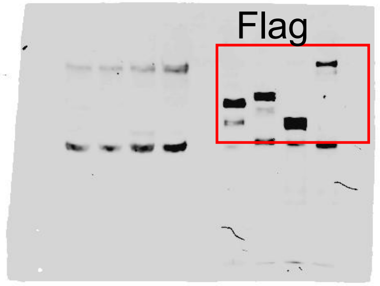

Flag

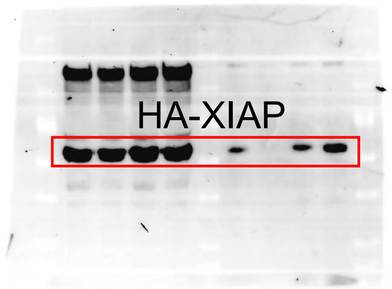

HA-XIAP

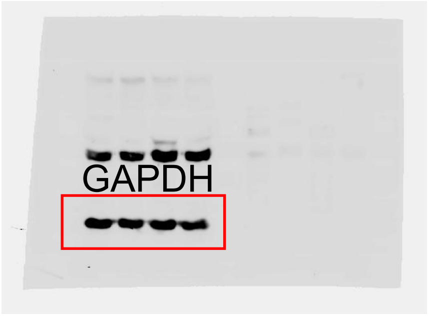

GAPDH

Figure 5H

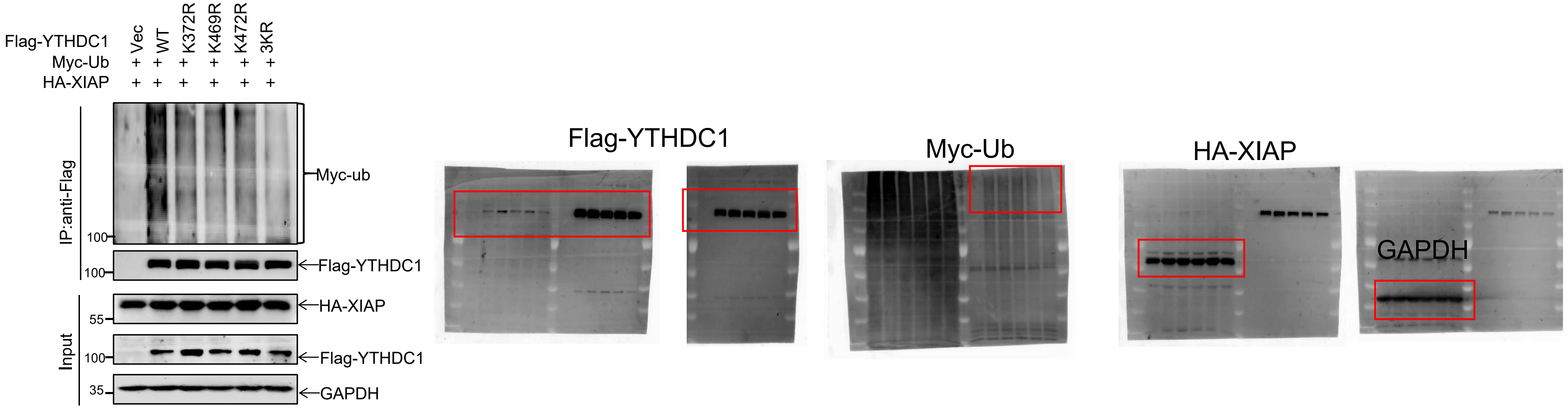

Figure 5I

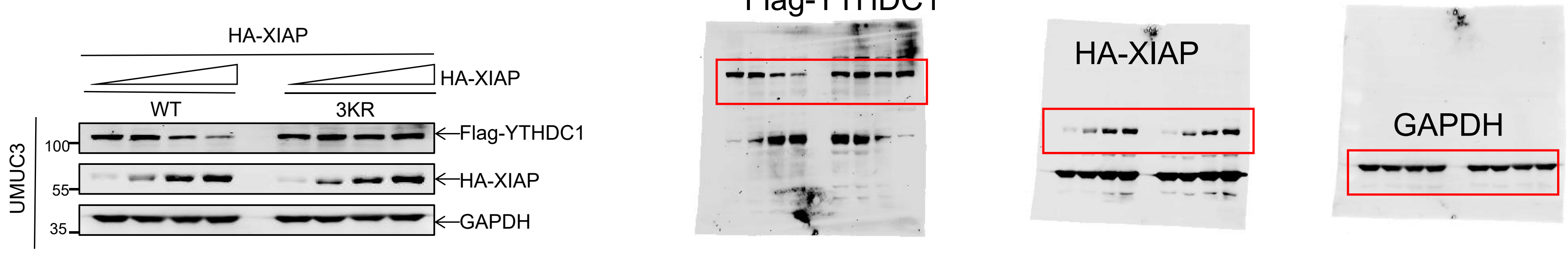

Figure 6A

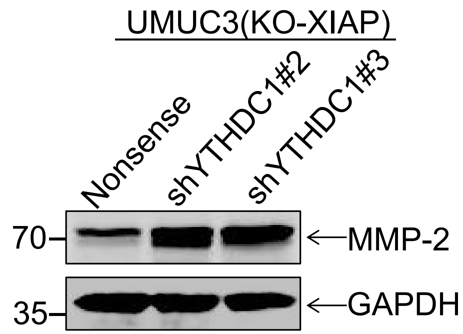

Figure 6B

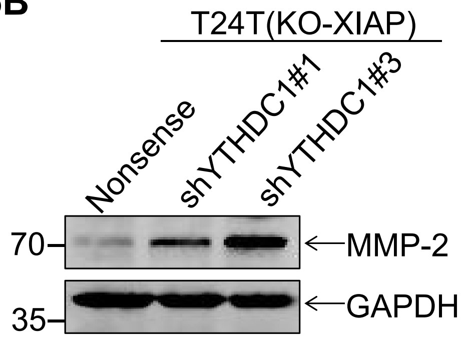

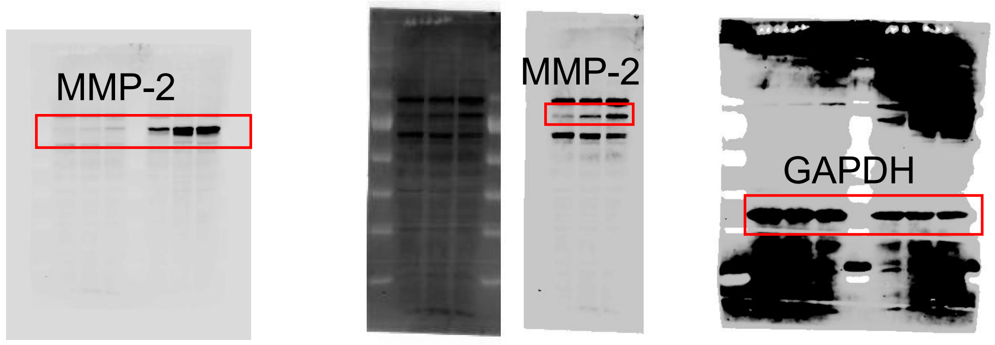

Figure 6C

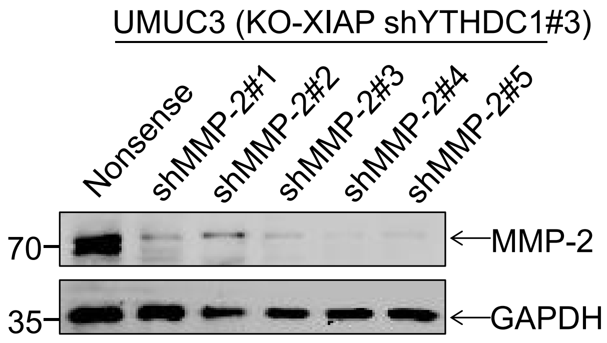

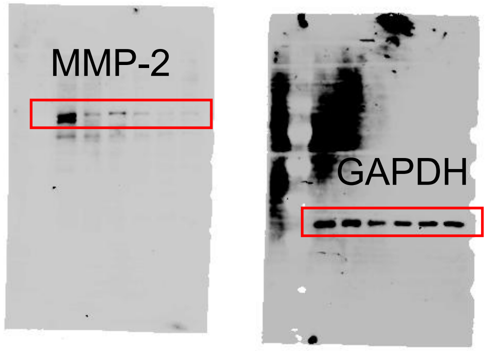

Figure S2A

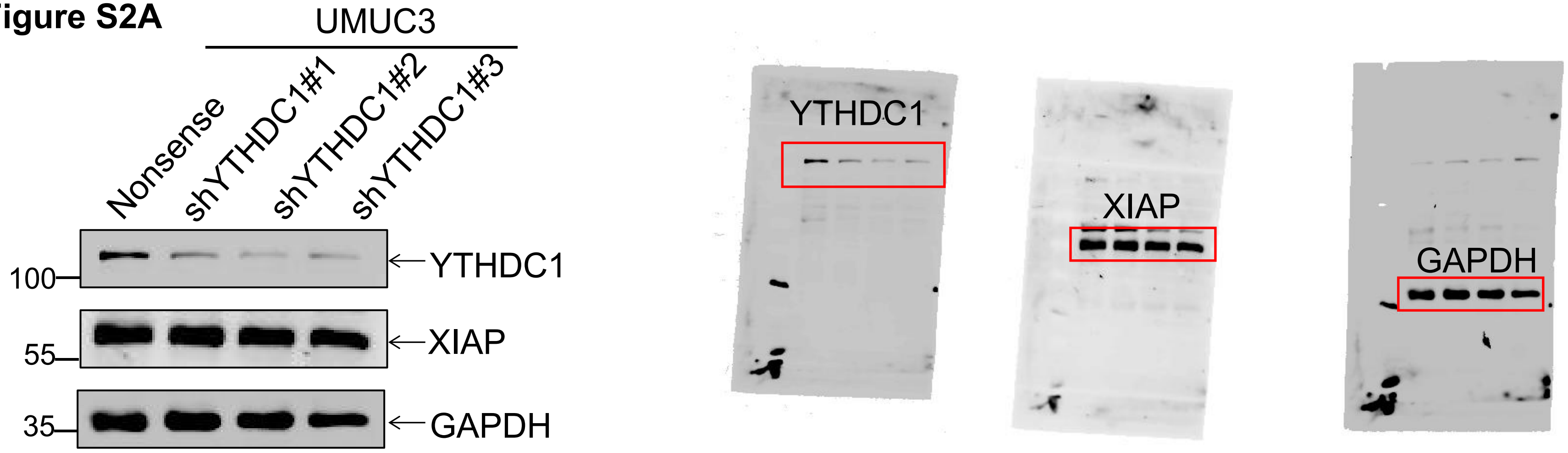

Figure S2B

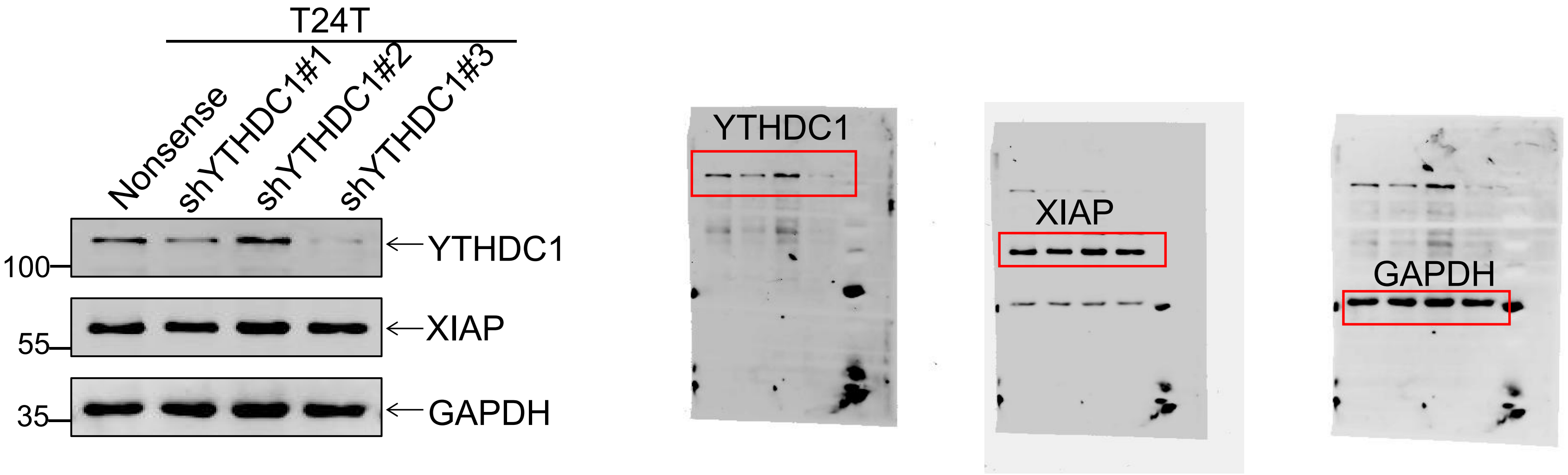

Figure S4

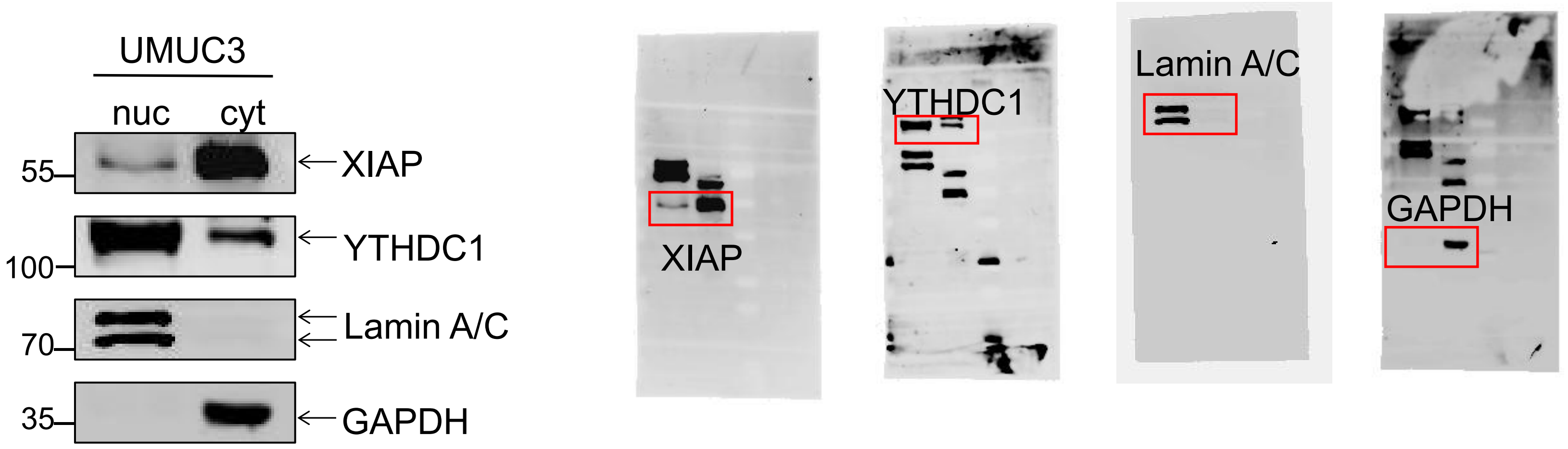

Figure S5

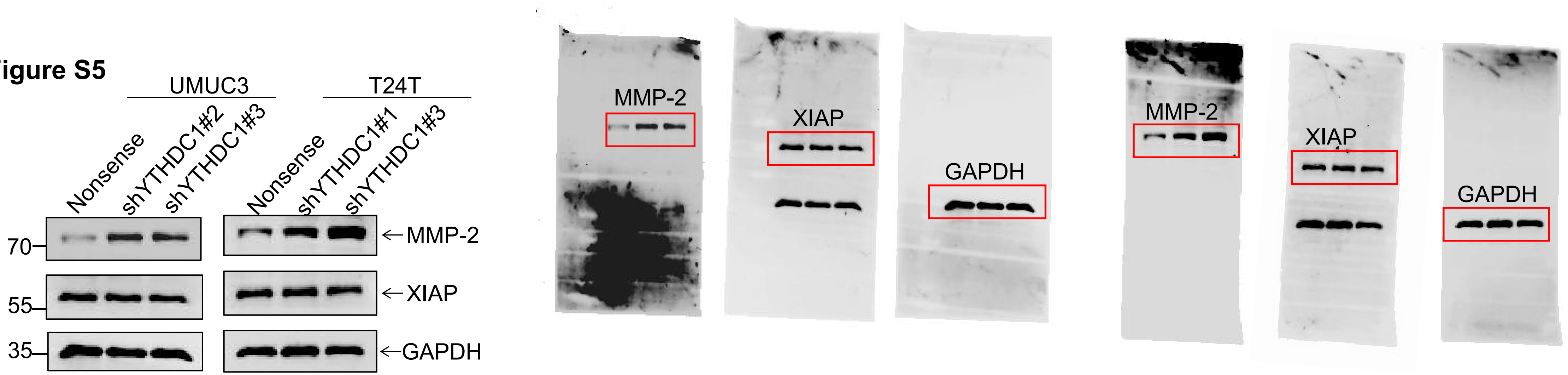

Figure S6A

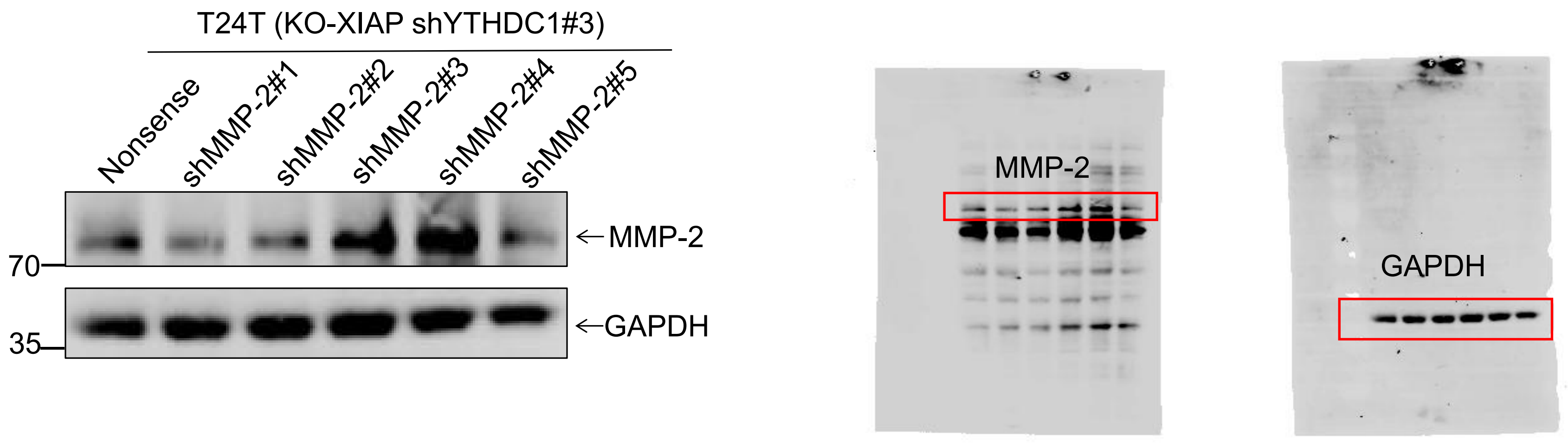

Figure S7A

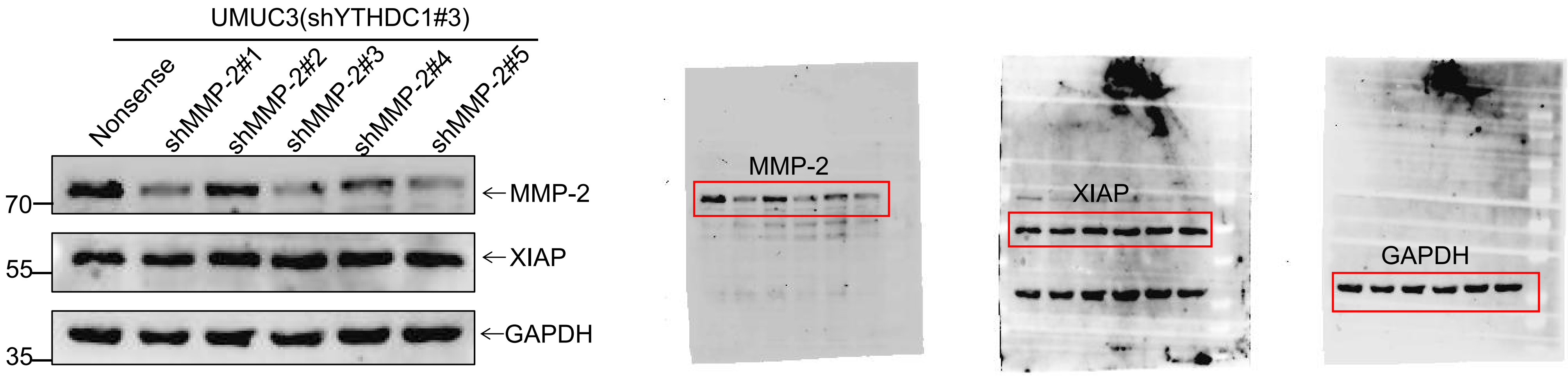

Supplement: Supplementary file 2 — Original western blots [file 41419_2025_7545_MOESM2_ESM.pdf]
